# Motor learning leverages coordinated low-frequency cortico-basal ganglia activity to optimize motor preparation in humans with Parkinson’s disease

**DOI:** 10.3389/fnins.2025.1542493

**Published:** 2025-05-13

**Authors:** Kara N. Presbrey, Thomas A. Wozny, Kenneth H. Louie, Simon Little, Philip A. Starr, Reza Abbasi-Asl, Doris D. Wang

**Affiliations:** ^1^Department of Neurological Surgery, University of California San Francisco, San Francisco, CA, United States; ^2^Department of Neurology, University of California San Francisco, San Francisco, CA, United States; ^3^Weill Institute for Neurosciences, University of California San Francisco, San Francisco, CA, United States

**Keywords:** Parkinson’s disease, movement decoding, functional connectivity, sequencing, motor learning, motor planning, fine motor control, hierarchical networks

## Abstract

Learning dexterous motor sequences is crucial to autonomy and quality of life but can be altered in Parkinson’s disease (PD). Learning involves optimizing pre-movement planning (preplanning) of multiple sequence elements to reduce computational overhead during active movement. However, it is unclear which brain regions mediate preplanning or how this process evolves with learning. Recording cortico-basal ganglia field potentials during a multi-day typing task in four individuals with PD, we found evidence for network-wide multi-element preplanning that improved with learning, facilitated by functional connectivity. In both cortex and basal ganglia, pre-movement gamma (*γ*, 30–250 Hz) activity, historically linked to population spiking, distinguished between future action sequences and became increasingly predictive with learning. For motor cortex *γ*, this increase was tied to learning-related cross-frequency coupling led by cortically-driven network delta (*δ*, 0.5–4 Hz) synchrony. More generally, coordinated network *δ* supported a complex pattern of learning-driven cross-frequency couplings within and between cortex and basal ganglia, including striatal lead of cortical beta (*β*, 12–30 Hz) activity, reflecting the specialized roles of these brain regions in motor preparation. In contrast, impaired learning was characterized by practice-driven decreases in *γ*’s predictive value, limited cross-frequency coupling and absent network *δ* synchrony, with network dynamics possibly altered by pathologically high inter-basal ganglia *δ* synchrony. These results suggest that cortically-led *δ* phase coordination optimized cortico-basal ganglia multi-element preplanning through enhanced recruitment of higher-frequency neural activity. Neurostimulation that enhances cortico-basal ganglia *δ* synchrony may thus hold potential for improving skilled fine motor control in PD.

## Introduction

Fine motor control is a fundamental aspect of human motor function. Skilled hand movements often require learning a sequence of finger movements, and proficiency is vital to maintaining autonomy. In Parkinson’s disease (PD), progressive decline in fine motor sequence learning and control, not solely attributable to hallmark motor symptoms, detrimentally impacts quality of life, with needs unmet by conventional deep brain stimulation (DBS) and dopamine replacement therapy (Doyon, 2008; Wu et al., 2015; Marinelli et al., 2017; Vasu et al., 2024; Rahman et al., 2008; Lee et al., 2010; Foki et al., 2015; Vanbellingen et al., 2011; Gebhardt et al., 2008; Ramirez-Zamora and Ostrem, 2018; Eisinger et al., 2019; Muehlberg et al., 2023; Ingram et al., 2021; Teixeira and Alouche, 2007; Park, 2017; Heilman, 2020). This decline may be linked to dysfunction in motor preparation, so closed-loop DBS targeting pathological variations in preparatory neural activity could remediate symptoms (Kumari and Kouzani, 2021; Simmonds et al., 2008; Ariani and Diedrichsen, 2019; Ariani et al., 2021). However, the learning-dependent neural dynamics of fine motor sequence initiation are poorly understood.

Before the onset of rapid fine motor sequences like typing, humans can plan multiple sequence elements (multi-element preplanning), and learning involves the optimization of this process (Ariani and Diedrichsen, 2019; Ariani et al., 2021; Verwey et al., 2015; Wong et al., 2015; Verwey, 1994). For sequences composed of at least one differing element within the first few elements, this predicts sequence-specific pre-movement neural activity that is optimized with learning. Indeed, neurophysiology studies in rodents and nonhuman primates suggest that multi-element preplanning is facilitated by the sequence-specific serial activation of neurons in motor cortical and basal ganglia (BG) ensembles, with motor improvement partly driven by increased consistency of ensemble spiking patterns (Khanna et al., 2021; Ganguly and Carmena, 2009; Peters et al., 2014; Rostami et al., 2024; Guo et al., 2021; Ganguly et al., 2022; Carrillo-Reid, 2022; Lu and Ashe, 2005; Hatsopoulos et al., 2003). However, in humans, it is unknown which brain regions have sequence-specific pre-movement neural dynamics or how sequence-specific activity changes with learning.

The neural processes that promote consistent ensemble firing patterns with learning also remain unclear, but recent investigation highlights the potential role of pre-movement oscillatory network dynamics. Human studies suggest that network-wide beta (*β*) desynchronization enables an increase in motor cortical excitability—reflected by a shift to the excitatory phase of motor cortical delta (*δ*)—which facilitates the activation of motor cortical ensembles to initiate movement (Khanna et al., 2021; Ganguly et al., 2022; Hahn et al., 2019; Mazzoni et al., 2010; Schroeder and Lakatos, 2009; Lakatos et al., 2008; Muller et al., 2018; Whittingstall and Logothetis, 2009; Hamel-Thibault et al., 2018; Ferreri et al., 2014; Fasano et al., 2022; Brown, 2003; Meissner et al., 2019; Singh, 2018; Rockhill et al., 2023; Mäki and Ilmoniemi, 2010; Schutter and Hortensius, 2011; Berger et al., 2014; Bhatt et al., 2016; Kaufman et al., 2014; Churchland and Shenoy, 2024). Work in animal models suggests that learning-driven corticostriatal *δ* synchrony enhances *δ*-ensemble spike coupling in striatum and motor cortex, resulting in the consistent ensemble firing patterns associated with motor improvement (Khanna et al., 2021; Ganguly et al., 2022; Lemke et al., 2019). Learning-driven changes in *β*’s influence of cortical excitability could also support motor improvement. However, these proposed network interactions have not been tested with cortico-basal ganglia electrophysiology and directed connectivity analysis in humans or animal models.

**Figure 1:**
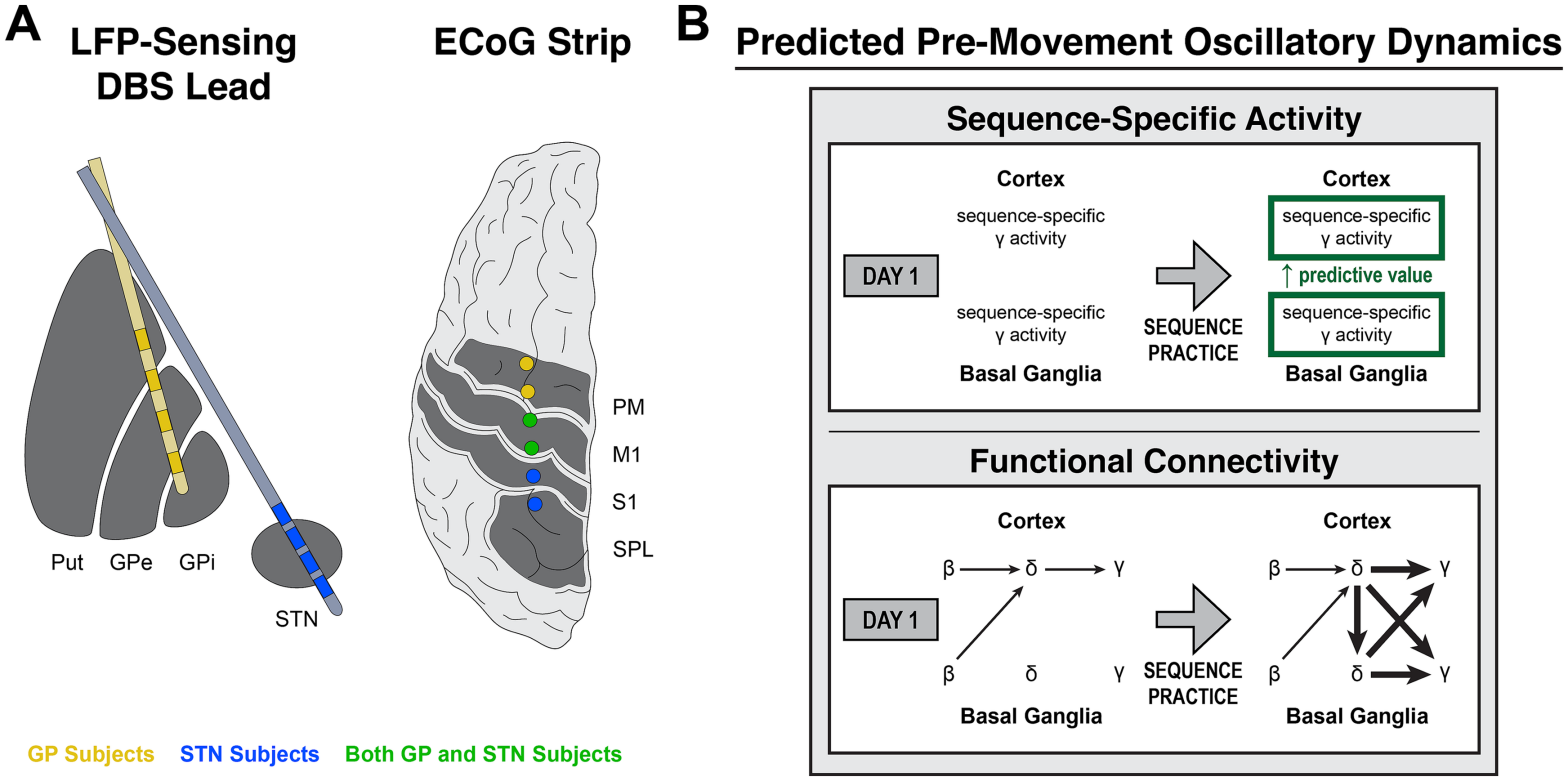
Predicted learning-related cortico-basal ganglia activity during motor sequence initiation. **(A)** Illustration of lead targeting for subject groups (LFP, local field potential; DBS, deep brain stimulation; ECoG, electrocorticography; Put, putamen; GPe, globus pallidus externus; GPi, globus pallidus internus; STN, subthalamic nucleus; PM, premotor cortex; M1, primary motor cortex; S1, primary somatosensory cortex; SPL, superior parietal lobule). **(B)** Diagram of predicted learning-related changes in sequence-specific activity and functional connectivity prior to the onset of motor sequences over multiple days of practice. (Top) For rapid, sequential finger movements, learning involves the optimization of preplanning for multiple sequence elements (multi-element preplanning), potentially implemented by increased reliability of sequence-specific ensemble firing patterns in cortex and basal ganglia. As *γ* activity correlates with population spiking, this could be reflected by sequence-specific *γ* activity that becomes increasingly predictive of future action sequences with practice. (Bottom) Oscillatory network dynamics are thought to drive general motor initiation and may display learning-dependent changes that lead to the increased reliability of ensemble activity patterns associated with motor improvement. One possibility is that network *β* desynchronization enables increased motor cortical excitability, reflected as a shift to the excitatory phase of cortical *δ*. In turn, excitability facilitates activation of motor cortical ensembles to produce movement, reflected by increasing *γ* amplitude. With motor learning, increased cortico-basal ganglia *δ* synchrony facilitates enhanced ensemble recruitment in cortex and basal ganglia, reflected by *δ*-*γ* coupling. We thus predicted the presence of network *β* → cortical *δ* → cortical *γ* interactions on all days and that, with practice, increased cortico-basal ganglia *δ* synchrony would accompany *δ*-*γ* coupling in cortex and basal ganglia.

We postulated that motor cortex and basal ganglia regions all support multi-element preplanning in PD, which network activity optimizes with successful learning. To test this, we evaluated the learning-dependent preparatory motor control network dynamics in four individuals with PD. We recorded cortico-basal ganglia field potentials while subjects performed a multi-day, multi-sequence typing task (Figure 1A). We hypothesized that *β* → *δ* → spike interactions influence motor cortex regardless of learning stage but that, with practice, coordinated cortico-basal ganglia *δ* activity increases the consistency of sequence-specific cortical and basal ganglia spiking patterns through *δ* → spike coupling. Field potential gamma (*γ*) activity correlates with neural population firing (Chang, 2015). Thus, *γ* activity could reflect temporal patterns and variability in ensemble activity. This anticipates specific *β* → *δ* → *γ* interactions, as well as sequence-specific motor cortex and basal ganglia *γ* activity that is increasingly predictive of future action sequences with learning (Figure 1B). We tested these predictions using single-trial classification of neural activity and directed connectivity analysis.

## Methods

### Study criteria

**Figure 2:**
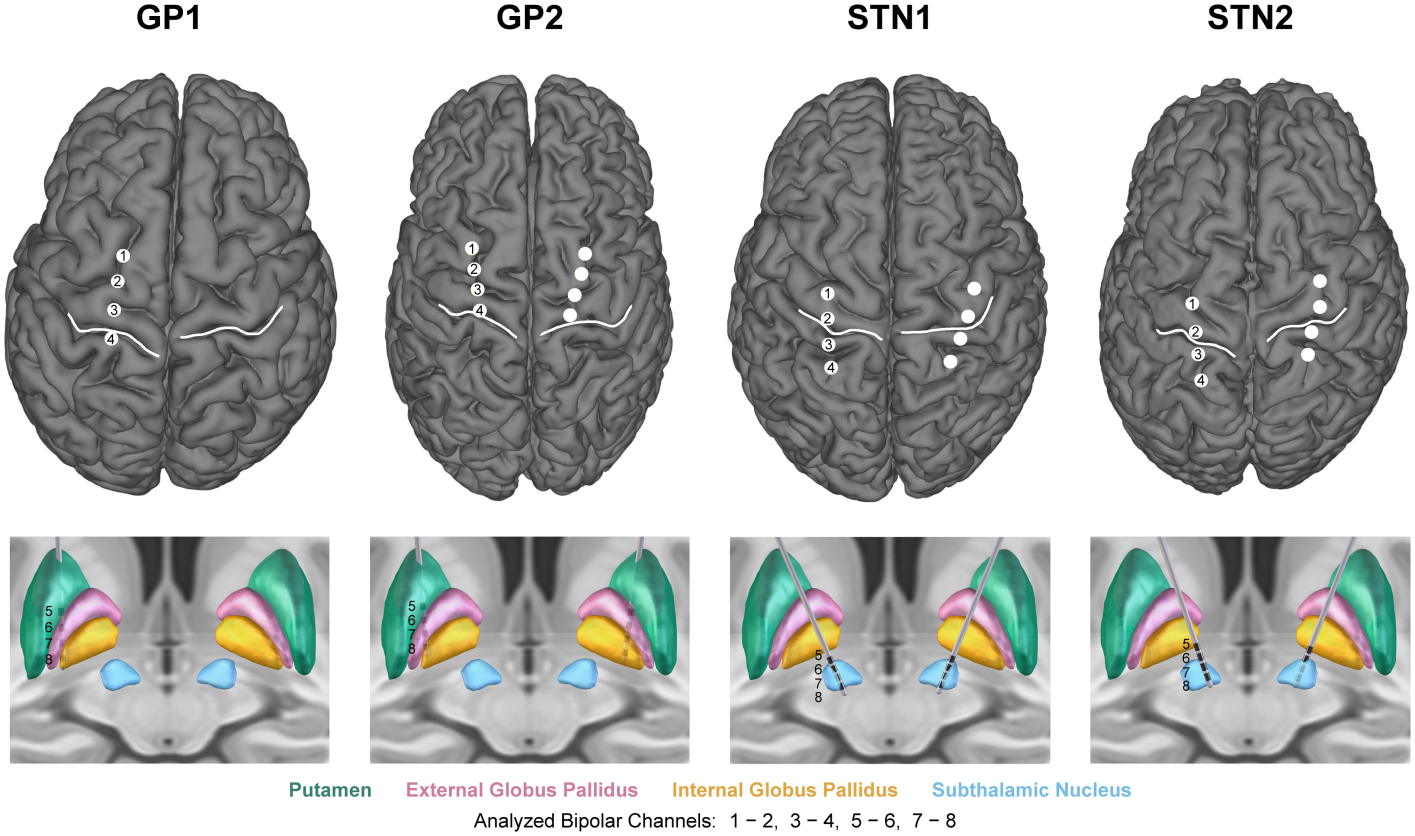
Cortical and subcortical lead reconstructions. (Top) Sensorimotor quadripolar electrocorticography strips, central sulcus (white), and (Bottom) quadripolar deep brain stimulation leads localized within the basal ganglia. Left hemisphere neural data (contralateral to movement), recorded using the specified bipolar montages, were used for neural analysis.

Four individuals enrolled in parent clinical trials (NCT03582891 and NCT04675398) for adaptive deep brain stimulation (DBS) for Parkinson’s disease (PD) participated in this study (Figure 2; Supplementary Table 1). Subjects had sufficiently severe movement disorder symptoms, inadequately treated by oral medication, and requested surgical intervention. No subjects exhibited significant untreated depression, significant cognitive impairment, previous cranial surgery, drug or alcohol abuse, or evidence of a psychogenic movement disorder. For an exhaustive list of overarching clinical trial inclusion and exclusion criteria, see NCT03582891 and NCT04675398. Additional prescreening was performed for the typing task. Inclusion criterion: enthusiastic desire to participate in the task. Exclusion criteria: hand or wrist pain when typing, dyslexia, uncorrected visual impairment, sleep apnea, travel to other time zones in the past 3 months. Subjects were also instructed not to consume nicotine or alcohol for the duration of the experiment. Subjects gave informed consent, and the University of California San Francisco Institutional Review Board pre-approved experimental design.

### Task design

**Figure 3:**
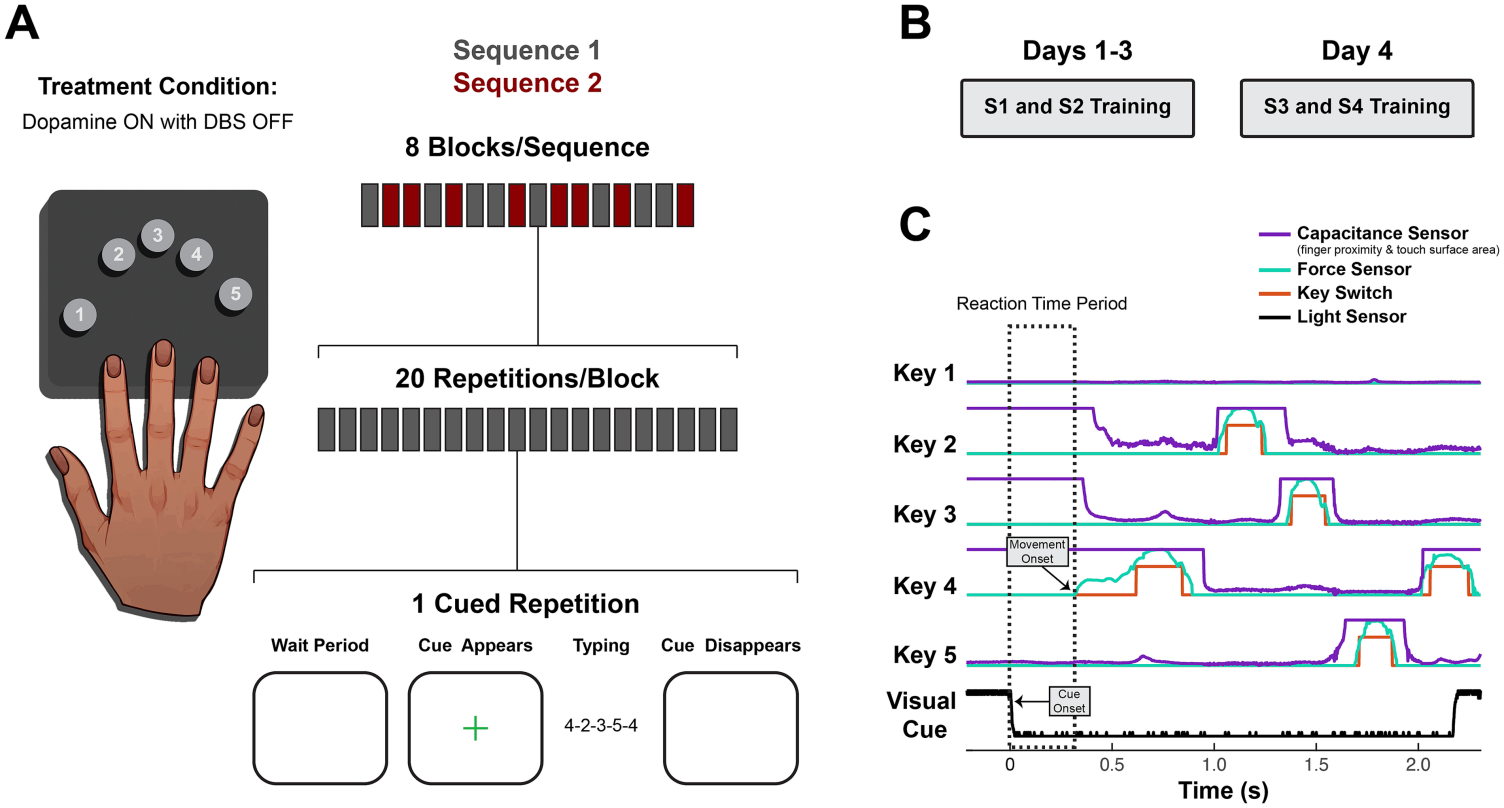
Experimental design and behavioral data collection. **(A)** On each day, subjects practiced typing two sequences. Interleaved practice blocks each contained 20 repetitions of visually cued sequence production for a single sequence. At the start of each block, the sequence was briefly shown and removed. A green fixation cross cued each subsequent trial. Subjects performed the task while on dopamine medication, and no DBS was delivered during the task or between days. **(B)** Days 1–3 employed novel Sequences 1 and 2 (S1 and S2), and Day 4 employed novel Sequences 3 and 4 (S3 and S4). **(C)** The reaction time period (dashed box) used for neural analysis is demonstrated in an example trial showing raw data from a custom behavioral setup used to capture finger movement (using capacitive proximity/touch sensors, force-sensitive resistors and mechanical key switches) and the visual cue (using a photodiode placed on the task computer screen). Capacitance sensors were calibrated to detect proximity changes of fingers hovering 0 to 2 cm above the keys (capacitance variation around low values). They could also detect changes in surface area of finger contact with the key associated with changes in force subthreshold for the force sensors (capacitance variation between low/mid-range and ceiling values). Thus, capacitance sensor readings were used for motor onset detection, except when motor onset began with a finger already in full contact with the key, in which case force sensor readings were used (as in the first keypress of this example trial).

On each day of a multi-day explicit motor learning experiment, subjects practiced typing two 5-element sequences in interleaved blocks using their dominant (right) hand while neural activity was recorded from the contralateral hemisphere (Figure 3A). The task design within each day was a variation of the common discrete sequence production task (Abrahamse et al., 2013). At the start of each session, subjects memorized that day’s sequences during an initial Verification Period. In this Verification Period, they were briefly shown one sequence to memorize before repeatedly typing it from memory until achieving three consecutive fully correct repetitions. This was repeated with the second sequence. Subjects were then instructed to, in the subsequent training blocks, react as quickly as possible and type as quickly and accurately as possible. In each training block, they practiced only one sequence. They typed one sequence repetition from memory in response to each cue. Each practice block started with its own Verification Period for the sequence for that block. The sequence was not shown again in that block. Green go cues appeared after an exponentially jittered delay from the 5th keypress of the previous trial (range: 0.85 s–3.75 s, *μ* = 1.75, *p* = 0.4 for Lilliefors test for h0 = exponential). A 10 s break followed each block. Subjects’ hands and the keypad were completely visually occluded, and the sequences were never displayed during typing. At the end of the task each day, subjects were assessed on the upper limb component of the Movement Disorder Society Unified Parkinson’s Disease Rating Scale (MDS-UPDRS). The day before the experiment, subjects were familiarized with the task and keypad with a practice run-through (Familiarization).

They received two novel sequences on Day 1 and again on Day 4 (Figure 3B; Supplementary Table 2). No sequences contained repeated adjacent elements, rising or falling triplets, or the thumb. All sequences paired for comparison within and between days started with the same first and last elements.

All subjects performed the experiment within 1 month after DBS surgery, before turning on DBS. No DBS was delivered during or between experimental sessions. To limit the effect of medication-related motor fluctuations, all experimental sessions were conducted at a consistent time across days within each subject’s medication ON period.

### Data collection

GP1 received a unilateral (left hemisphere) neural implant; all other participants received bilateral implants. In each implanted brain hemisphere, a four-contact DBS lead spanned basal ganglia (BG) nuclei, and a four-contact electrocorticography (ECoG) paddle spanned sensorimotor cortex (Figure 2). Bipolar recording of subcortical local field potentials (LFPs) and sensorimotor electrocorticography (ECoG) signal granted coverage of the following approximate regions in the left (contralateral) brain hemisphere. Subjects GP1 and GP2: globus pallidus (GP), putamen (Put), M1 or primary sensorimotor cortex (M1/S1), premotor cortex. Subjects STN1 and STN2: ventral subthalamic nucleus (vSTN), dorsal subthalamic nucleus (dSTN), M1, parietal cortex (spanning S1 and superior parietal lobule).

Leads from each brain hemisphere (Medtronic 3,387 for globus pallidus, 3,389 for subthalamic nucleus and 0913025 for cortex) were connected to a bidirectional neural interface in the ipsilateral chest (Medtronic Summit RC + S B35300R). LFP and ECoG signals were recorded at 500 Hz throughout the task. Channels were referenced to the metal casing of the implanted pulse generator. On-device hardware low and high pass filtered the data at 450 Hz and 0.85 Hz, amplified it, then performed another low pass filter at 1700 Hz.

Task events and keystroke data were captured in 4-kHz sweeps using a portable custom-made device run by a Teensy 4.1 microcontroller (Figure 3C), which acted as the master clock and motherboard for a custom keypad, visual stimulus detector and electrical impulse detector. To ensure accurate detection of finger movement onset/offset, even when finger position started above but not touching the key, a combination of custom capacitive proximity sensors (carved from copper sheet metal, 3DDeluxe), force-sensitive resistors (FSRs, Alpha MF01A-N-221-A04) and linear mechanical key switches (CHERRY MX1A-LxxA/B) were used for each digit. An FSR was fixed atop each custom keycap (3DDeluxe).

A small resin disk with a centered bulge less than a millimeter tall was fixed atop each FSR. This ensured even that off-center finger contact with the key face would result in force distribution to the FSR’s center active zone sufficient to drive detectable FSR activity. The resin disks also insulated the FSRs from the proximity sensors, which were cut from copper sheet metal and fixed atop each resin disk. To maximize proximity sensor read rate, each proximity sensor was sampled by its own Teensy 3.2 microcontroller, which each transmitted readings to the Teensy 4.1. Proximity sensors were covered with insulating tape and calibrated to detect proximity changes of fingers hovering up to ~2 cm above the keys. The capacitance sensors also detected changes in surface area of finger contact with the key. This enabled detection of changes in finger contact slight enough that the associated change in force was subthreshold for the force sensors. A photodiode (Everlight Electronics Co Ltd., PD333-3C/H0/L2) fixed to the task computer screen captured the timing of visual stimuli and progression of experimental epochs. For neural-behavioral data stream alignment, a unique temporal pattern of fifteen single DBS pulses was delivered at the start and end of each experimental session and detected along the metal casing of the pulse generator by an external electrical signal detector (MikroElectronika EEG Click MIKROE-3359). All sensors were calibrated and checked for electrical interference and cross-talk at the start of each experimental session.

### Behavioral analysis

To eliminate outlier trials, we excluded incorrect trials and any trials with a reaction time (RT, cue onset to movement onset) or trial duration (movement onset to offset) exceeding three standard deviations of the block average for correct trials.

To evaluate overall learning, we computed a block slowness-error index for each subject. Each block slowness-error index is the sum of block average error rate (1 – block accuracy), reaction time (cue presentation to movement initiation) and trial duration (movement onset to offset). For each subject, the block average trial durations and reaction times were each first min-max scaled to [0, 1], using data from all days to derive the minimum and maximum values. Lower slowness-error index values indicate better performance.

### Neural analysis

All significance testing for neural analysis utilized permutation testing that simulates error within the null distribution, and secondary tests were performed only to assess the direction of primary detected effects. Multiple comparison correction was therefore not performed.

#### Trial selection

In addition to the behavioral cutoffs applied for behavioral analysis, the following trial exclusion criteria and trial subsampling methods were performed for neural analysis. Trials with less than 25 ms between final/first movements associated with adjacent trials were excluded. Subsequently, subsampling was performed within a given day to match trial counts between sequences within each group of four blocks to avoid a possible imbalance over time, e.g., 75% of remaining Sequence 1 (S1) trials coming from the first half of the session and 75% of remaining Sequence 2 (S2) trials coming from the second half of the session. Within each group of four blocks, sequence subsampling followed epoch-specific selection rules. Only trials with RT ≥ 100 ms were considered. Trials from the higher count sequence were subsampled to match trials from the lower count sequence based on RT durations. Finally, random subsampling matched trial counts across days within each subject for each epoch type.

#### Signal preprocessing

Neural signal preprocessing used the following pipeline. Data from each channel was linearly detrended, demeaned and high-pass filtered at 0.25 Hz using a two-pass FIR filter. Electrical noise was excluded in the frequency domain. Two-pass Kaiser FIR filters with normalized transition widths of ≤ 0.1 were used for all subsequent bandpass filtering.

Data intended for single-trial classification, amplitude analysis and undirected phase analysis was filtered with the following passbands: *δ* (0.5–4 Hz), *θ* (4–8 Hz), *α* (8–12 Hz) and *β* (12–30 Hz for amplitude analysis and cross-frequency coupling, 12–20 Hz and 20–30 Hz for single trial classification). For *γ*, filters were logarithmically spaced from 30 to 250 Hz. High *γ* (70–250 Hz) center frequencies were used for all analyses involving *γ*, while slow (30–50 Hz) and mid (50–70 Hz) *γ* center frequencies were used only for single-trial classification. These filters were all non-overlapping in the frequency domain to reduce collinearity between adjacent frequency bands when performing single-trial classification. The Hilbert transform computed the analytic signal. For undirected cross-frequency coupling (CFC) analysis using pairwise phase consistency (PPC), the resulting amplitude envelope of each *β* and *γ* center frequency was filtered with the same bandpass filter previously used to extract *δ*, followed by a second application of the Hilbert transform.

For analysis of directed phase coherence (including directed CFC) using phase slope index (PSI), *δ* was instead extracted using linearly spaced passbands (0.5, 3.25; 0.75, 3.5; 1, 3.75; 1.25, 4). For directed *δ*-*β* and *δ*-*γ* coupling analysis, these linearly spaced *δ* passbands were applied to the *β* amplitude envelope and to the amplitude envelope of each center frequency of high *γ* (70–250 Hz), followed by a second application of the Hilbert transform.

For artifact screening, filter-Hilbert was used to estimate 70–250 Hz broadband amplitude, which was then *z*-scored over the entire session. Any trial in which *z* ever surpassed 8 standard deviations was omitted from neural analysis.

#### Single-trial classification

If two sequences contain at least one differing element within the first few elements, then multi-element preplanning necessitates some sequence-specific neural activity. To test for neural activity related to multi-element preplanning, we thus tested for sequence-specific preparatory neural dynamics using single-trial classification of pre-movement neural activity. To evaluate learning-driven optimization of multi-element preplanning, we then evaluated change in sequence-specific predictive value of neural activity with practice by testing change in model performance across days.

A different classifier was trained on data from each recording channel on each day, and mean decoding accuracy was used to estimate the discriminability of sequence-specific neural activity. S1- or S2-labeled trial data was extracted from the RT period in the *t_n_* ms immediately prior to sequential movement onset, where *t_n_* was the average RT on Day 3 for Subject *n*. Data then underwent feature selection and logistic classification with *L_1_* regularization. For each subject, trials per sequence were balanced across classes, channels and days.

Time-frequency regions with maximal differences between sequences were selected as features. To assess, e.g., differences in narrowband amplitude dynamics in PM for S1 vs. S2 on Day 3 for GP1, we calculated the two-sided *t-*statistic for amplitude at each time-frequency point. For each of 10,000 permutations, trial labels were shuffled, and the *t*-statistic was recalculated. Thus, each time-frequency point had an associated null distribution of 10,000 *t*-statistic values. Time-frequency points at which the test value fell below the 80th percentile compared to its respective null distribution were masked. In each of the remaining islands of features for each center frequency, the time point with the highest percentile score relative to its null was selected as a feature to use in the model. For all resulting features, corresponding amplitude values were taken from S1 and S2 trial data. For phase data, the same process was implemented, save for two differences. Phase opposition sum (VanRullen, 2016) was used instead of the *t*-statistic, and since phase is a circular process, each selected phase value was converted into two features: sin(phase) and cos(phase). Each amplitude and phase feature was median-centered and scaled according to its interquartile range to have unit variance.

Hyperparameter optimization, model training and model testing were performed with nested cross validation. The inverse *L_1_* regularization constant (*λ*^−1^) was optimized per classifier in 10-fold, 10-repeat stratified cross validation performed on a stratified 90% subset of the data. The following *λ*^−1^ values were tested: 5E-2, 1E-1, 5E-1, 1, 5, 1E1, 5E1, 1E2, 5E2, 1E3, 5E3, 1E4, 5E4, 1E5, 5E5. Greater shrinkage produced performance at or below chance level. The selected value for *λ*^−1^ was then used for final model training and testing on the full dataset with 10-fold, 100-repeat stratified cross validation.

Permutation testing evaluated for significant sequence-specific neural activity in each brain region and how the predictive value of neural activity changed with practice. Right-sided permutation testing assessed significance of model mean decoding accuracy relative to chance. The outer 10-fold, 100-repeat stratified cross validation was repeated 1,000 times with permuted trial labels, and the resulting 1,000 null values were compared to test sample mean decoding accuracy. Two-sided permutation testing assessed the change in mean decoding accuracy across days for a given channel. Mean decoding accuracies for each of the 100 repeats per day were permuted across days, and the between-day difference in overall mean decoding accuracy was recalculated for each permutation as the null value.

#### Feature importance testing

To evaluate how learning-driven changes in sequence-specific neural activity might be reflected in the spectral characteristics of field potential recordings, the absolute importance of various signal properties to the performance of trained models was tested and compared across days. We permuted, in the test set, the trial labels for all phase *or* amplitude features associated with a given canonical frequency band, as the majority of across-frequency or across-phase/amplitude feature correlations were not high (*ρ* < 0.5). For each fold in each repeat of the 10-fold 100-repeat outer cross-validation used for prior model training and testing, the test data trial labels for the respective trained model were permuted once for a given feature group, and the resulting change in test accuracy from test performance was computed. Change in decoding accuracy was then averaged across all 10 folds in each of the 100 repeats. Feature groups for which permutation produced a negligible accuracy increase were set to zero in data plots for visual clarity. No instance of accuracy increase with feature permutation surpassed 1%.

We then repeated group permutation testing, except with all phase and amplitude features for *δ* through *β* grouped together and likewise for low *γ* through high *γ*. Change in decoding accuracy was averaged across folds per repeat before between-day permutation testing.

#### Coherence analysis

Functional connectivity was evaluated over the course of practice to assess which network interactions may support learning-driven optimization of multi-element preplanning. Single-trial plots indicated that *δ* phase aligned to cue in various regions, so data was aligned to cue and evaluated in a window length of the mean RT of Day 3 per subject. Nonparametric cluster-based permutation across time, with a cluster size correction, was used for all phase analyses. To simplify data visualization, significant across-day effects associated with low and insignificant levels of within-day local or interregional coherence were not typically depicted with shaded time regions in the figures, but the *p*-values are still reported in the Supplementary Data Tables.

For all phase analyses, we first computed each metric within sequence before averaging the resulting time series across sequences prior to statistical testing. This was intended to address two main issues. First, we expected possible sequence-specificity in spatiotemporal patterns of neural activity that could be reflected in mesoscale spectral activity—an idea for which both single-trial classification and single-trial *δ* phase plots then provided confirmatory evidence. This implies that different sequences could be associated with different characteristic *γ* amplitude envelope morphologies, which may display different phase-specific coupling patterns with *δ*. Second, in cases for which two sequences were not performance-matched on a given day (e.g., Sequences 3 and 4), one may observe differences in activity between sequences due to performance level (rather than learning stage). In either case, a reasonable approach would be to respect the sequence-specific relationships, so we first computed metrics within each sequence. However, we also expected the general oscillatory network dynamics associated with learning to be the same regardless of sequence, so we then averaged the resulting metric time series across sequences before statistical testing.

Inter-trial *δ* phase locking value (PLV) assessed cue-aligned consistency of local *δ* phase (Lachaux et al., 1999). For a given recording channel, the resulting time series (one for each sequence) were smoothed with a 150 ms-long Gaussian window and averaged across sequences. To test for significant PLV on a given day, we randomly sampled phase data from the duration of the session for each permutation. PLV was computed for each sequence null group using the appropriate number of trials for each sequence. The magnitudes of the resulting two null PLV time series were then smoothed and averaged across trial groups to attain a single null time series for that permutation. This was repeated for each permutation. To test for a significant difference in PLV time series between days, we calculated the test time series by subtracting the PLV time series from 1 day from that of the other day. For each permutation, trials were shuffled across days but within sequence.

Inter-trial *δ* pairwise phase consistency assessed cue-aligned interregional *δ* phase coherence (Vinck et al., 2010). For each channel pair, the resulting time series (one for each sequence) were smoothed with a 150 ms-long Gaussian window and averaged across sequences. For baseline PPC testing, methods were identical to those used in baseline PLV testing, except the null was constructed by sampling channel data as pairs, i.e., the baseline distribution corresponded to an actual estimate of baseline session-wide coherence for that channel pair, not to the level of coherence that would be expected if the two channels were coupled only randomly. For between-day PPC permutation testing, test time series were calculated by subtracting the PPC time series between days, and for each permutation, phase data for both channels in a given channel pair were shuffled together across days but within sequence.

To assess the direction of pairwise *δ* phase relationships observed with PPC, phase slope index between two channels was computed per time point for each sequence (Nolte et al., 2008). PSI values were not smoothed before being averaged across sequences. To assess whether significant *δ* phase lead/lag occurred with respect to chance, i.e., neither channel led the other, rather than with respect to session baseline, data for each permutation was randomly sampled from the session duration separately for each channel in the pair, for each sequence.

Pairwise phase consistency and phase slope index were also used to estimate undirected and directed coherence, respectively, for cross-frequency couplings. *δ* Phase was paired with the *δ* phase of the *β* or *γ* amplitude envelope. Computations analogous to those used for *δ* synchrony analysis were performed, except for two modifications. For *δ*-high *γ* coupling, PPC or PSI was calculated separately for each narrowband within 70–250 Hz for each sequence. The result was averaged across *γ* center frequencies before averaging across sequences. Second, for baseline permutation testing, the phase data was held constant while the amplitude data was sampled from the session. Shuffling amplitude while holding phase constant was intended to test for significant coherence *given* a specific phase distribution.

## Results

### Behavioral stratification based on sequence learning

**Figure 4:**
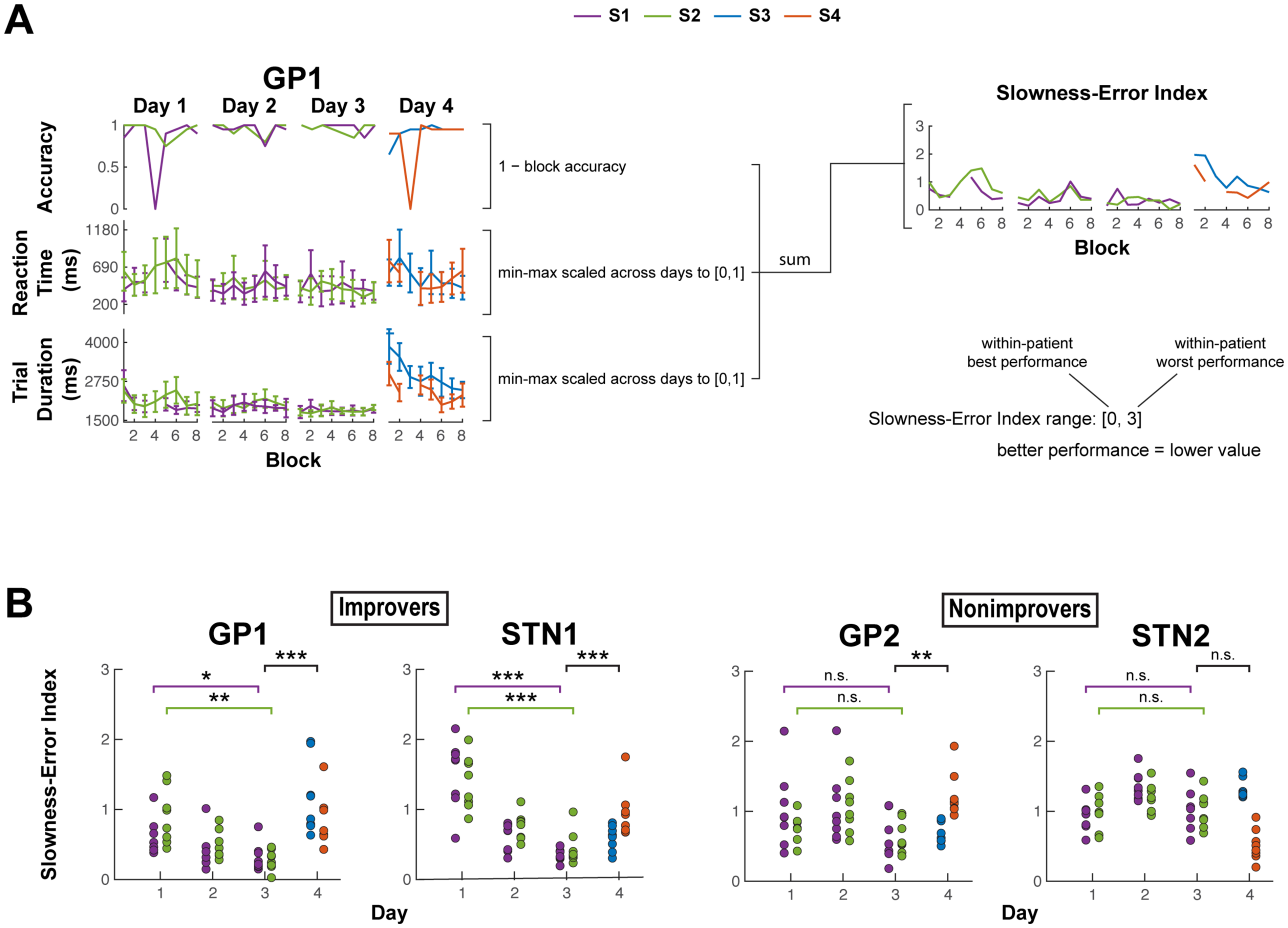
Behaviorally distinguishing improvers and nonimprovers. **(A)** Example calculation of block slowness-error index from block average data. Each block slowness-error index is the sum of block average error rate [1 – accuracy], reaction time [cue onset to movement onset] and trial duration [movement onset to offset]. For each subject, the block average trial durations and reaction times were each first min-max scaled to [0, 1], using data from all days to derive the minimum and maximum values. Error bars indicate ± *s*. **(B)** Block slowness-error index for each subject. Comparison across days 1 and 3 for each of S1 and S2 assessed within-sequence practice-driven performance changes. To help evaluate whether performance changes from day 1 to day 3 were at least in part related to sequence learning and not solely attributable to changing familiarity with the task and keypad, performance was also compared between pooled day 3 sequences and pooled day 4 sequences. Subjects were labeled improvers (ID ending in 1) only if their performance both improved from day 1 to day 3 and worsened when presented with novel sequences on Day 4 (For all comparisons: *α =* 0.05, two-sided, two-sample *t*-test with unequal variance. For within-sequence comparisons and pooled sequence comparisons, *n* = 8 and 16 sequence blocks per group, respectively, except for GP1 day 1 S1 and GP1 Day 4 for which *n* = 7 and 15 due to exclusion of 0% accuracy blocks, as composite performance would be poorly defined). * *p* < 0.05, ** *p* < 0.01, *** *p* < 0.001. S1–S4, Sequence 1–4.

Subjects were behaviorally stratified for sequence learning based on changes in a composite measure of block performance—a block slowness-error index—for which lower value corresponds to better performance (Figure 4A). Day 1 to Day 3 comparisons of pooled block performance indices for each of S1 and S2 evaluated within-sequence practice-driven performance changes. Significant decrease in slowness-error index suggests sequence learning, but improvement on S1 and S2 could also have been driven by more general task learning, e.g., optimization of task-related cognitive processes and motor familiarization with the experimental apparatus (Ariani and Diedrichsen, 2019; Taylor and Ivry, 2011; Wulf, 2013; Song, 2019; Clark and Ivry, 2010; Serrien et al., 2007; Doyon and Benali, 2005). Even so, superior performance of S1 and S2 on Day 3 compared to that upon subsequent presentation of novel sequences on Day 4 would suggest some sequence learning had in fact occurred for S1 and S2. Thus, subjects were labeled improvers (ID ending in 1) only if their performance both improved from Day 1 to Day 3 and worsened when presented with novel sequences on Day 4. A Day 1 to Day 4 comparison to assess general task learning is confounded by behavioral interference between the familiar sequences and those presented on Day 4, so we did not attempt to behaviorally stratify based on task learning.

GP1 and STN1 showed indications of sequence learning, whereas GP2 and STN2 did not (Figure 4B; Supplementary Figures 1, 2). In GP1 (S1: *p* = 0.032; S2: *p* = 0.002) and STN1 (S1: *p* < 0.001; S2: *p* < 0.001), slowness-error index improved for both sequences from Day 1 to Day 3, then worsened when practicing novel sequences on Day 4 (GP1: *p* < 0.001; STN1: *p* < 0.001). Neither GP2 nor STN2 showed significant improvements in Sequence 1 (S1) and Sequence 2 (S2) slowness-error index (*p* > 0.05 for all), and only GP2’s slowness-error index significantly differed between Days 3 and 4 (GP2: *p* = 0.005; STN2: *p* > 0.05). These results suggest behavioral stratification as follows: improvers (GP1 and STN1) and nonimprovers (GP2 and STN2).

Sleep durations and end-of-session upper limb scores on the Movement Disorders Society Unified Parkinson’s Disease Rating Scale (MDS-UPDRS) were similar between groups (Goetz et al., 2008) (Supplementary Table 3). This suggests relative differences in performance improvement may not have been due to large differences in sleep or motor symptom presentation in the typing arm.

### Pre-movement cortical and basal ganglia *γ* activity is sequence-specific and increasingly predictive of sequence content with performance improvement

**Figure 5:**
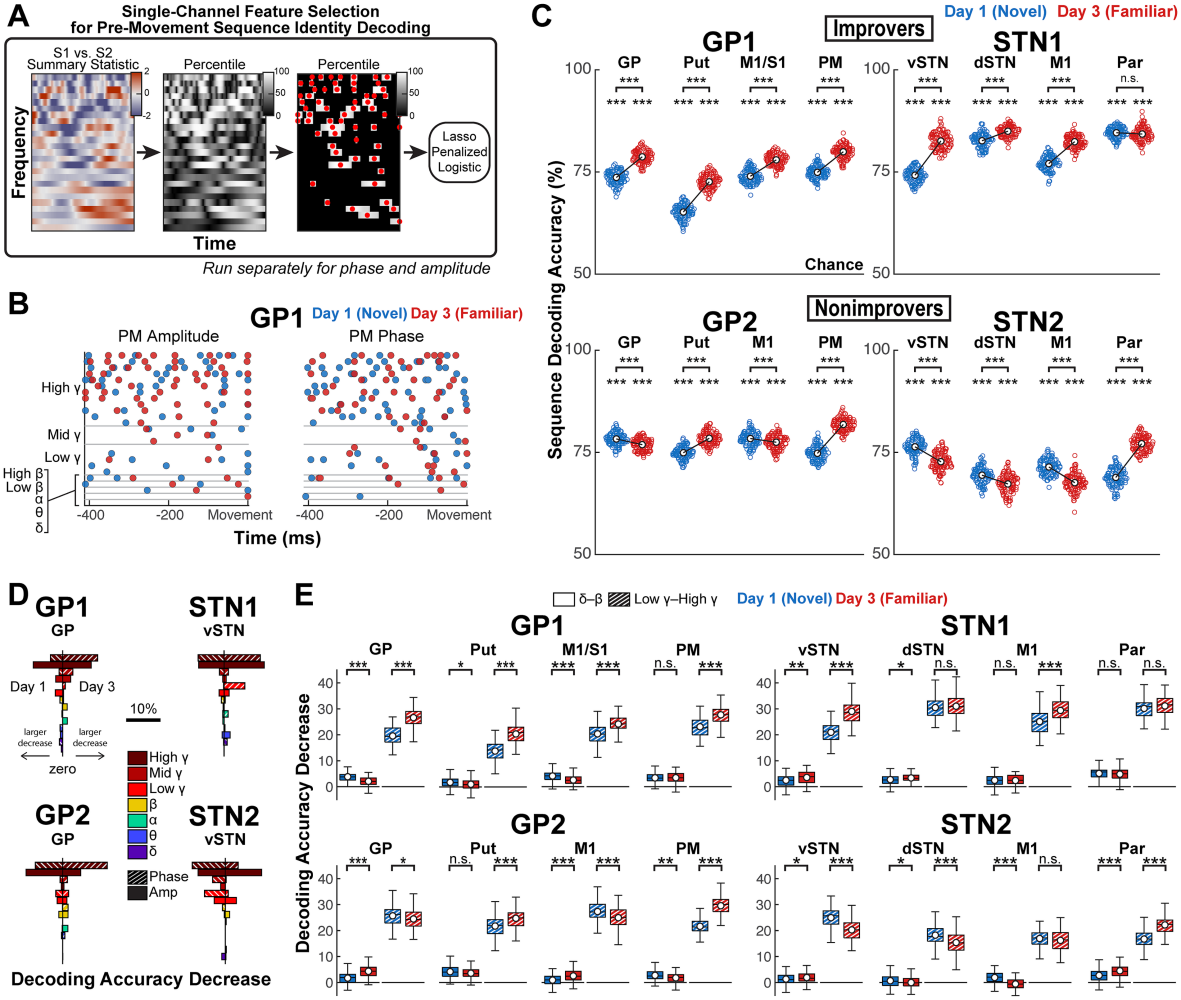
Pre-movement sequence-specific *γ* activity, present in all brain regions, demonstrates practice-driven increases and decreases in discriminability in improvers and nonimprovers, respectively. **(A)** Visualization of feature selection pipeline. Neural activity prior to the onset of sequential movement was selected to predict the identity of the sequence the subject was about to type. Feature selection was repeated for separate models for each channel on each day in each subject. (Left) For selection of amplitude features, the S1 vs. S2 two-sided *t*-statistic was computed at each time-frequency point. (Middle) The *t*-statistic at each time-frequency point was recomputed for 10,000 permutations of trial labels to determine the percentile ranking of the test value at each time-frequency point relative to its null distribution. (Right) Time-frequency points falling below their respective 80th-percentile cutoffs were masked, and in each of the remaining time-frequency regions, the time point achieving the highest percentile was selected as an amplitude feature. This process was repeated for phase data, using phase opposition sum as the summary statistic. Each resulting phase feature was split into two features that corresponded to the cartesian phase coordinates. Classification utilized 10-fold 100-repeat lasso-penalized logistic classification. **(B)** Example selected features for Days 1 and 3 in GP1’s PM. **(C)** Mean decoding accuracy per model after feature selection (Comparison to chance: *α =* 0.05, one-sided, permutation testing with 1,000 resamples). Comparison across days: *α =* 0.05, two-sided, permutation test with 10,000 resamples. Empty circle reflects mean decoding accuracy across folds for one repeat; white circle reflects mean decoding accuracy across repeats. In **(D,E)**, grouped feature permutation testing was performed to test the importance of different features to model performance. **(D)** Decreases in absolute decoding accuracy with permutation of features grouped by canonical frequency band and signal property (amplitude or phase). Impact on model performance of shuffling each feature group is shown for Day 1 (left of vertical line) and Day 3 (right of vertical line) in a subset of representative brain regions. **(E)** Decreases in absolute decoding accuracy with permutation of features instead grouped only by frequency and into two groups: *δ* through *β* (0.5–30 Hz, phase and amplitude) and low *γ* through high *γ* (30–250 Hz, phase and amplitude) (Comparison across days: *α =* 0.05, two-sided, permutation testing with 10,000 resamples). White circle reflects mean; black horizontal line reflects median. Box edges correspond to 25th and 75th percentiles. Whiskers span the entire data range excluding outliers. Outliers were computed as 1.5·*IQR* away from the upper or lower quartile and are not shown. For all analysis in this figure, *n* sequence trials per day = 238 for GP1, 206 for GP2, 158 for STN1, 182 for STN2. * *p* < 0.05, ** *p* < 0.01, *** *p* < 0.001. GP, globus pallidus; Put, putamen; vSTN, ventral subthalamic nucleus; dSTN, dorsal subthalamic nucleus; M1, primary motor cortex; PM, premotor cortex; S1, primary somatosensory cortex; Par, parietal cortex.

We next assessed which brain regions might participate in multi-element preplanning and how this changes with learning. We used single-trial classification of pre-movement neural activity to predict the identity of the upcoming sequence. Model performance thus quantified sequence-specific predictive value of neural activity. For each experiment day and each recording channel per subject, we performed feature selection on neural data preceding sequential movement onset and trained a model to predict the identity of the sequence that the subject was about to type (Figures 5A,B; Supplementary Figure 3). To isolate practice-driven changes in sequence-specific neural activity from changes that occur as a byproduct of the changing behavior, we only directly compared neural activity between sequences for which overall behavioral performance was similar. In most subjects, performance levels significantly differed between Sequences 3 and 4 (Days 1–3: *p* > 0.05 in all subjects; Day 4: *p* > 0.05 in GP1, *p* = 0.002 in GP2, *p* = 0.031 in STN1, *p* < 0.001 in STN2) (Supplementary Figure 4), so Day 4 data was excluded from single-trial classification analysis. To reduce the influence of neural activity related to the first sequence element, we designated the same digit as the first element in all sequences for each subject. See *Methods: Neural analysis* for additional measures taken to reduce the influence of confounds.

We compared each model’s performance to chance and tested within-channel change in decoding accuracy across Days 1 and 3, with the caveat that Day 1 to Day 3 changes may reflect effects of both sequence practice and task exposure. Notably, sequence-specific activity was detected throughout the recorded network in all subjects (*p* < 0.001 for all models) (Figure 5C). Practice drove nearly network-wide increases of this activity’s predictive value in improvers (*p* < 0.001 for all except Par; STN1 Par: *p* > 0.05) but decreases in GP (*p* < 0.001), STN (*p* < 0.001 for vSTN and dSTN) and M1 (*p* < 0.001 for GP2 and STN2) in nonimprovers (significant increase in nonimprovers’ other channels with *p* < 0.001).

To assess which electrophysiological signal properties granted sequence-specific predictive value, we performed feature analysis. Minimal correlations (*ρ* < 0.5) between features grouped by phase, amplitude and canonical frequency band allowed grouped feature permutation testing (Supplementary Figure 5). Low *γ* (30–50 Hz), mid *γ* (50–70 Hz) and high *γ* (70–250 Hz) were collectively the most important, though the importance of *γ* phase relative to and *γ* amplitude varied (Figure 5D; Supplementary Figure 6). Thus, for formal statistical analysis, we divided features into only two groups: (1) *δ* through *β* and (2) low *γ* through high *γ* and found that *γ* activity was the primary driver of practice-driven changes in model performance (Figure 5E; Supplementary Figure 7). In most brain regions, the across-day change in accuracy decrease linked to permutation of grouped *γ* features was large and in a direction that paralleled practice-driven change in model performance (GP1: *p* < 0.001 in all brain regions; GP2: *p* = 0.025 in GP, *p* < 0.001 otherwise; STN1: *p* < 0.001 in vSTN and M1, *p* > 0.05 in dSTN and Par; STN2: *p* > 0.05 in M1, *p* < 0.001 otherwise). In contrast, effects for permutation of grouped *δ* through *β* features were small and did not consistently track model performance across days (GP1: *p* < 0.001 in GP and M1/S1, *p* = 0.013 in Put, *p* > 0.05 in PM; GP2: *p* < 0.001 in GP and M1, *p* > 0.05 in Put, *p* = 0.001 in PM; STN1: *p* = 0.004 in vSTN, *p* = 0.020 in dSTN, *p* > 0.05 in M1 and Par; STN2: *p* = 0.049995 in vSTN, *p* = 0.024 in dSTN, *p* < 0.001 in M1 and Par). These findings suggest network-wide participation in multi-element preplanning through sequence-specific population activity that was optimized with learning and reflected in *γ* activity.

### Improvement is associated with cortically-led *δ* phase synchrony in response to cue

We next evaluated how oscillatory network dynamics may have facilitated the observed learning-related changes in sequence-specific activity. Assuming that the overall architecture of functional connectivity is sequence-general even for sequence-specific spatiotemporal patterns of neural activity, we calculated functional connectivity for each sequence and averaged the result across sequences per day before statistical testing. This minimized confounds due to performance differences between learning stage-matched sequences. Thus, we could analyze all experimental sessions, and across-day comparisons of functional connectivity paralleled behavioral stratification. Sequence practice-related effects were those that occurred in overlapping time regions between *both* Days 1 and 3 and Days 3 and 4; though we did not behaviorally stratify task learning, we did examine task exposure-related neural activity by comparing Days 1 and 4.

**Figure 6:**
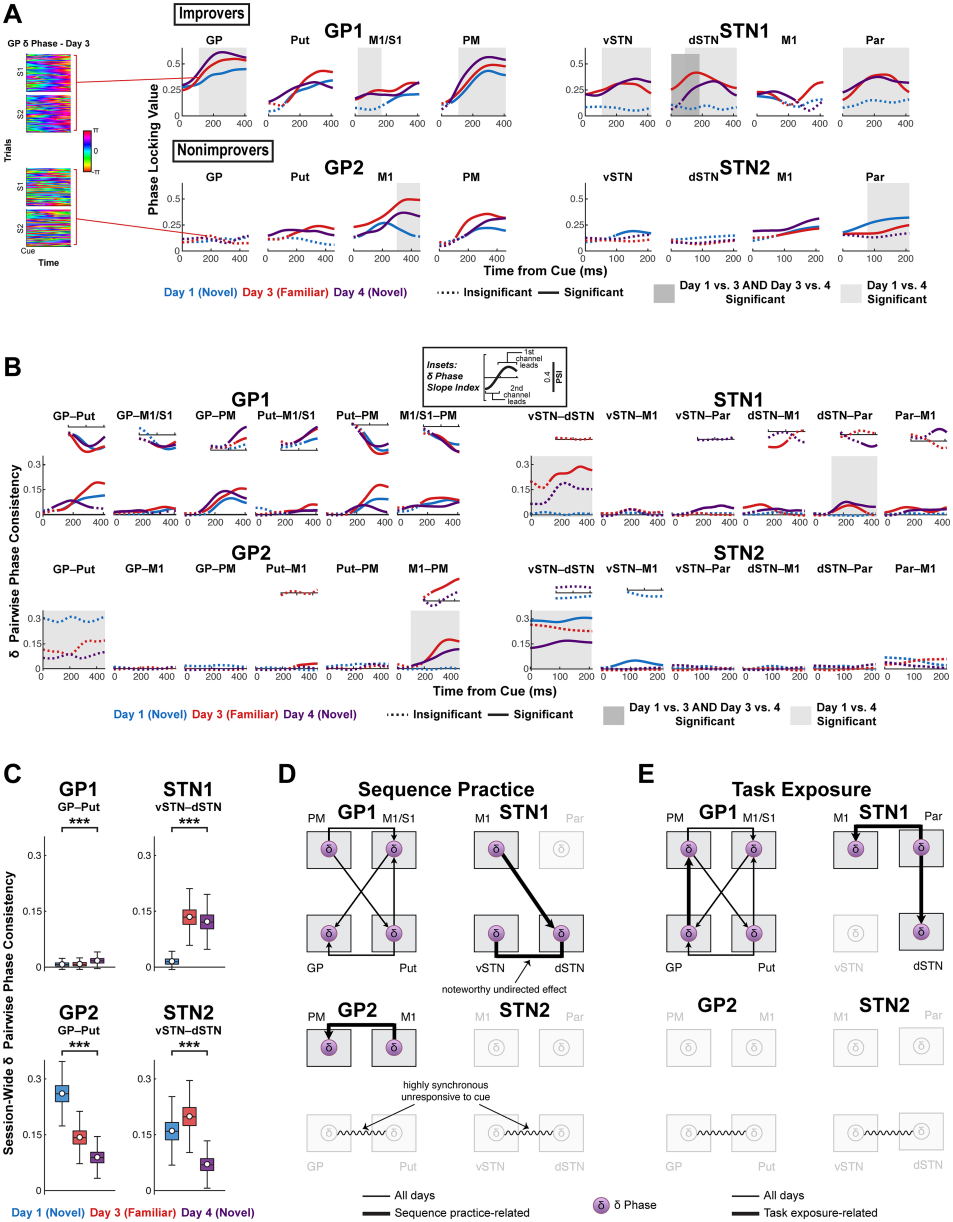
Improvement is associated with cortically-led network *δ* phase synchrony, to which sequence learning and task exposure add distinct effects, while lack of improvement is associated with highly synchronous BG *δ*. **(A)** (Left) All Day 3 single-trial *δ* phase time series after cue onset in the pallidum of GP1 and GP2. (Right) *δ* Phase locking value (PLV) computed across trials per channel after cue onset on days 1, 3 and 4. Solid line indicates PLV significantly higher than chance (α = 0.05, one-sided, cluster-based permutation test with 10,000 resamples). See Supplementary Table 4 for *p*-values. Shaded box indicates significant difference in PLV between days (α = 0.05, two-sided, cluster-based permutation with 10,000 resamples). See Supplementary Table 5 for *p*-values. **(B)** Effects in *δ* synchrony. (Large plots) *δ* pairwise phase consistency (PPC, undirected measure) time series for all channel pairs. Solid line indicates PPC significantly higher than session-wide baseline, i.e., *h_0_* = coherence aligned cue is the same as general coherence levels not aligned to cue (rather than *h_0_* = no coherence) (α = 0.05, one-sided, cluster-based permutation with 10,000 resamples). See Supplementary Table 6 for *p*-values. Shaded box indicates significant difference in PPC between days (α = 0.05, two-sided, cluster-based permutation with 10,000 resamples). See Supplementary Table 7 for *p*-values. (Insets) Phase slope index (PSI, directed measure) for data series in which PPC significantly surpassed baseline. PSI is displayed for the same total time window as PLV and PPC. Solid line indicates significant PSI (*h_0_* = no channel leads, α = 0.05, two-sided, cluster-based permutation with 10,000 resamples). See Supplementary Table 8 for *p*-values. **(C)** Session-wide baseline basal ganglia *δ* pairwise phase consistency averaged across time. Calculated by taking the null distribution of time series resampled from each session in **(B)** and averaging each null PPC sample across time. Change in session baseline across Days 1 and 4 was tested (α = 0.05, two-sided, permutation testing with 10,000 resamples). See Supplementary Table 9 for *p*-values. White circle reflects mean; black horizontal line reflects median. Box edges correspond to 25th and 75th percentiles. Whiskers span entire data range excluding outliers. Outliers were computed as 1.5·*IQR* away from the upper or lower quartile and are not shown. ***p < 0.001. **(D)** Network diagrams illustrating sequence practice-related *δ* coherence effects. **(E)** Network diagrams illustrating task exposure-related *δ* coherence effects. GP, globus pallidus; Put, putamen; vSTN, ventral subthalamic nucleus; dSTN, dorsal subthalamic nucleus; M1, primary motor cortex; PM, premotor cortex; S1, primary somatosensory cortex; Par, parietal cortex.

The supposition that *δ* phase facilitates recruitment of sequence-specific ensemble activity implies *δ* phase-spike coding, which ultimately predicts that *δ* phase consistently aligns to motor events. Cortical *δ* phase locks to movement-related visual cues in healthy subjects, so we computed *δ* phase locking value (PLV) to cue in our subjects after confirming all recording channels had *δ* amplitude sufficient for phase estimation (Lakatos et al., 2008; Hamel-Thibault et al., 2018; Lachaux et al., 1999; Saleh et al., 2010) (Figure 6A; Supplementary Figures 8, 9). In GP subjects, PLV was significant throughout motor cortex on all days, increasing with task exposure in M1 (or M1/S1). However, only GP1 had significant PLV in basal ganglia on all days and increasing PLV in GP and PM with task exposure. In STN subjects, M1 PLV was also significant on all days. In other channels (not surgically targeted in GP subjects), PLV effects differed between STN1 and STN2. In STN1, PLV increased with task exposure in vSTN and Par and with both sequence practice and task exposure in dSTN—effects absent in STN2, for whom PLV was mostly insignificant or diminished with task exposure. These results suggest that cue-aligned *δ* phase in motor cortex, striatum and, increasingly, dSTN was important to sequence learning and reveal an enhancement of motor network-wide *δ* phase alignment to cue with task exposure that occurred only in improvers.

Such anatomically widespread *δ* phase alignment to cue in improvers suggests cross-area coordinated *δ* activity sufficient to facilitate coordinated recruitment of motor cortical and striatal ensembles with learning (Ganguly et al., 2022). To assess this, we analyzed interregional *δ* phase coupling. Using pairwise phase consistency (PPC), we tested for an *increase*, relative to session-wide baseline, in undirected phase coherence aligned to cue and compared PPC between days (Vinck et al., 2010). When undirected coherence was significant, we compared the phase slope index (PSI; directed coherence) to chance (no channel leads) (Nolte et al., 2008). Two brain regions with a common input could show significant and stable undirected coherence even if a phase lead developed with learning. Undirected coherence exceeded baseline too infrequently to justify systematic between-day PSI testing, so we also noted as sequence practice- or task exposure-related any directed coherence that occurred only on specific days (Day 3 for sequence practice; Days 3 and 4 or Day 4 for task exposure).

Only improvers demonstrated cortically-led network *δ* synchrony (Figures 6B,D–E). This synchrony increased above session-wide baseline in response to the cue and demonstrated sequence learning- and task exposure-related effects in improvers. In GP1, PM led M1/S1 and Put, and Put led GP, with small but significant M1/S1-striatal coherence associated with leads for M1/S1 → GP and Put → M1/S1. GP led PM with task exposure. These effects were absent in GP2, for whom M1 instead led PM for familiar sequences. In STN1, M1 led dSTN for familiar sequences, and Par led dSTN and M1 with task exposure. Significant cue-related inter-STN PPC for familiar sequences indicates STN was recruited to network *δ* synchrony, partly by M1 and perhaps by an unrecorded region. In STN2, no directed phase leads occurred, and significant inter-STN PPC lacked the consistent local *δ* phase needed for event-locked coordinated phase coding (Figure 6A). Notably, in both nonimprovers, inter-BG *δ* synchrony started high and decreased with task exposure—an effect paralleled in session-wide inter-BG *δ* coherence, opposite that observed in improvers (Figure 6C). High inter-basal ganglia *δ* synchrony in nonimprovers was linked to a lack of cue-aligned local BG *δ* phase and an absence of cortico-basal ganglia *δ* synchrony.

### Sequence learning is associated with *δ*-*γ* coupling within and between cortex and basal ganglia

Having identified coordinated *δ* activity theoretically capable of supporting *δ* → spike coupling to facilitate the sequence-specific activity reflected in classification analysis, we next directly evaluated *δ*-*γ* coupling. We assessed both local and cross-area *δ* phase-high *γ* coupling. Using pairwise phase consistency and phase slope index, we calculated undirected and directed coherence between *δ* phase and the *δ* phase of the *γ* amplitude envelope (*γ*_h_*^δ^*). For interregional cross-frequency coupling (CFC), we report all results for which at least one subject demonstrates an effect of either sequence practice or task exposure.

Contrary to expectation, local *δ* → *γ*_h_*^δ^* coupling was rare, suggesting that local *δ* → spike coupling may not have been a primary mechanism facilitating sequence-specific activity (Supplementary Figure 10). Practice-dependent *γ*_h_*^δ^* → *δ* coupling was more common, though also inconsistent across subjects. Nonetheless, M1 high *γ* amplitude still significantly increased in the RT period on all days in all subjects (Supplementary Figure 11), as expected for successful movement. This indicates that activity reflected in local *δ* → *γ*_h_*^δ^* coupling was not a necessary step in general motor initiation for these subjects (Singh, 2018; Crone et al., 1998).

**Figure 7:**
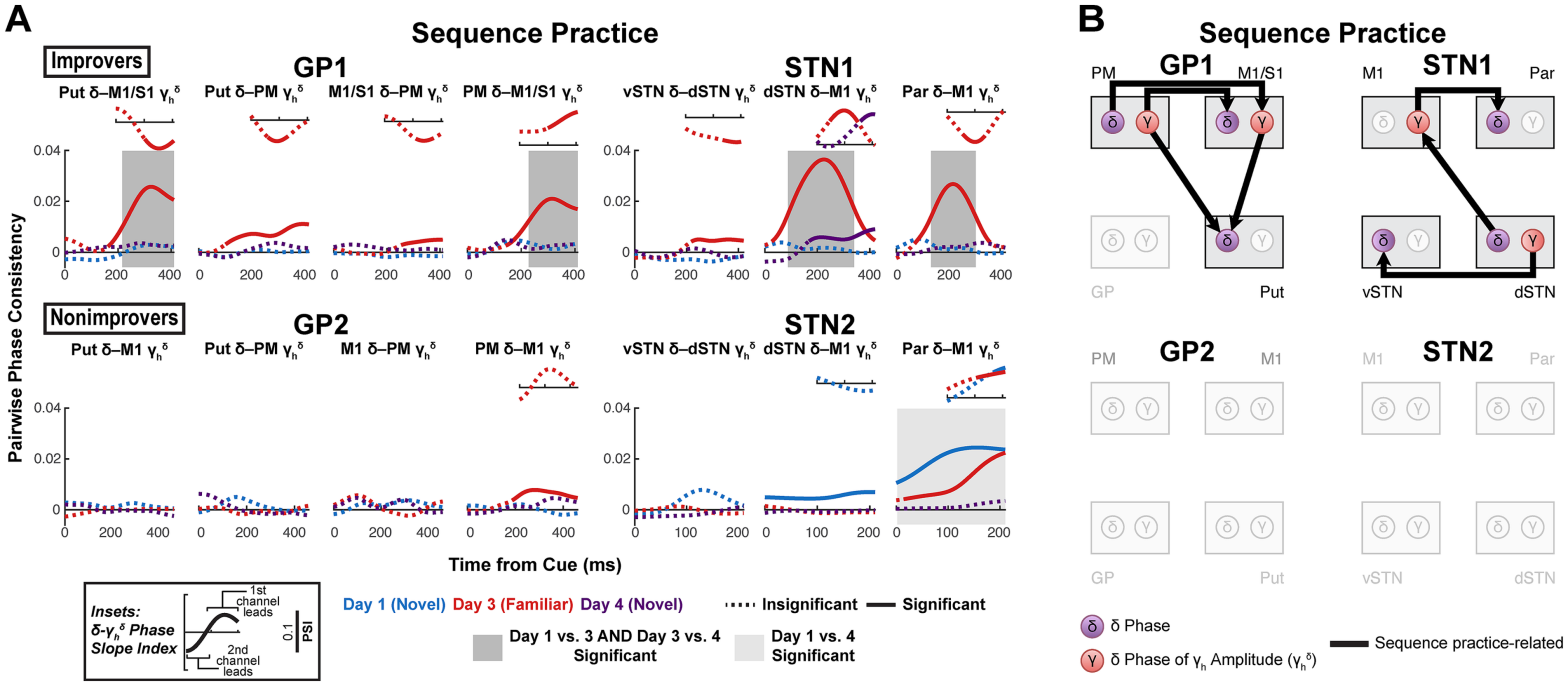
Sequence learning is associated with corticocortical, cortico-basal ganglia and inter-basal ganglia *δ*-*γ* couplings. **(A)** Sequence practice-related effects in interregional *δ*-*γ*_h_*^δ^* coherence. (Large plots) Pairwise phase consistency (PPC, undirected measure) was calculated between *δ* phase and the *δ* phase of the high *γ* amplitude envelope. Solid line indicates significant PPC (*h_0_* = coherence is not higher than expected given the phase distribution, *α =* 0.05, one-sided, cluster-based permutation with 10,000 resamples). See Supplementary Table 14 for *p*-values. Shaded box indicates significant difference in PPC between days (*α =* 0.05, two-sided, cluster-based permutation with 10,000 resamples). See Supplementary Table 15 for *p*-values. (Insets) Phase slope index (PSI, directed measure) for data series in which PPC was significant. PSI is displayed for the same total time window as PPC. Solid line indicates significant PSI (*h_0_* = no channel leads, *α =* 0.05, two-sided, cluster-based permutation with 10,000 resamples). See Supplementary Table 16 for *p*-values. **(B)** Network diagrams illustrating sequence practice-related *δ*-*γ*_h_*^δ^* effects. GP, globus pallidus; Put, putamen; vSTN, ventral subthalamic nucleus; dSTN, dorsal subthalamic nucleus; M1, primary motor cortex; PM, premotor cortex; S1, primary somatosensory cortex; Par, parietal cortex.

Sequence learning was more associated with a range of interregional *δ*-*γ*_h_*^δ^* effects (Figures 7A,B). These effects differed depending on the involved brain regions, which is consistent with the specialized roles of different regions in the cortico-basal ganglia network. In GP1 but not GP2, sequence practice was associated with premotor lead of M1/S1, as well as M1/S1 and PM lead of putamen. In STN1 but not STN2, sequence practice was associated with dSTN lead of M1 and vSTN, as well as M1 lead of Par. In STN2, Par instead led M1 on Days 1 and 3, but this effect diminished with task exposure. Otherwise, there were no task exposure-related effects in any subjects. Notably, *δ* led *γ*_h_*^δ^* only for M1 *γ*_h_*^δ^* with sequence learning in improvers, potentially reflecting underlying *δ* → spike coupling that led to the improvement-related increase in M1 *γ*’s predictive value (Figure 5).

### Network *β* does not gate motor cortical *δ*

Using the same approach, we finally tested whether network *β* gated motor cortical excitability by assessing *δ*-*β* coupling. We calculated coupling between *δ* phase and the *δ* phase of the *β* amplitude envelope (*β^δ^*). Cortical excitability reflected as a deflection in cortical *δ*, could be led by cortical *β*, subcortical *β* or a combination of both. Thus, we assessed both intraregional and interregional *δ*-*β^δ^* coupling.

There was substantial overlap of regions with consistent movement-related *β* desynchronization and regions with cue-related *δ* phase alignment (all motor regions in improvers; motor cortex and putamen in nonimprovers) (Figure 6A; Supplementary Figure 12), suggesting widespread local *δ*-*β* coupling. However, significant local *δ*-*β^δ^* directed coupling was rare, never occurred across all days and demonstrated inconsistent direction of phase lead in M1 across subjects (Supplementary Figure 13). Local *β* gating of low-frequency shifts in M1 excitability was not likely a general mechanism in motor initiation.

**Figure 8:**
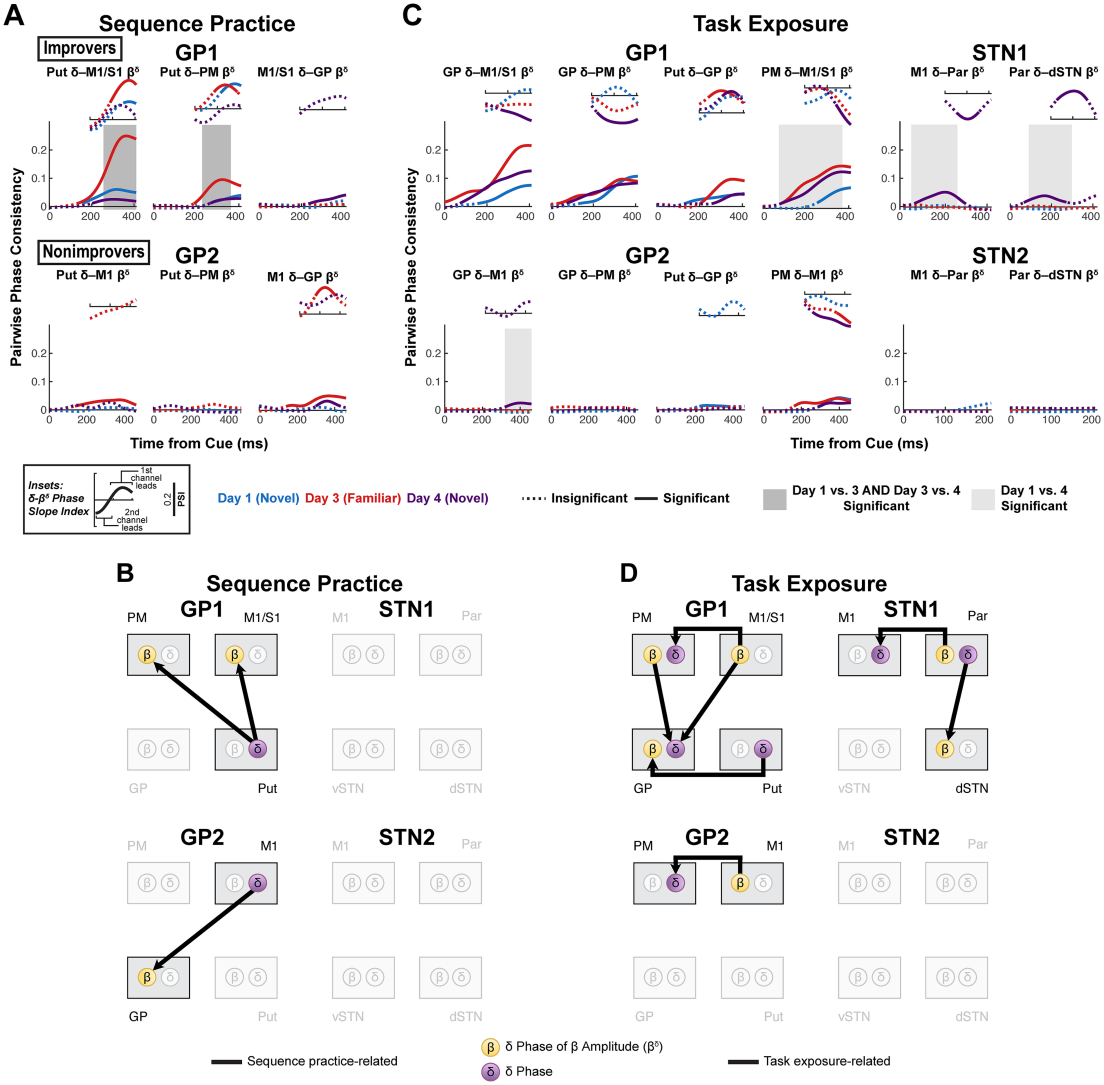
Striatocortical *δ*-*β* coupling increases with sequence learning, while task exposure brings a range of *δ*-*β* couplings in improvers mostly absent in nonimprovers. **(A)** Sequence practice-related effects in interregional *δ*-*β^δ^* coherence. (Large plots) Pairwise phase consistency (PPC, undirected measure) was calculated between *δ* phase and the *δ* phase of the *β* amplitude envelope. Solid line indicates significant PPC (*h_0_* = coherence is not higher than expected given the phase distribution, *α =* 0.05, one-sided, cluster-based permutation with 10,000 resamples). See Supplementary Table 21 for *p*-values. Shaded box indicates significant difference in PPC between days (*α =* 0.05, two-sided, cluster-based permutation with 10,000 resamples). See Supplementary Table 22 for *p*-values. (Insets) Phase slope index (PSI, directed measure) for data series in which PPC was significant. PSI is displayed for the same total time window as PPC. Solid line indicates significant PSI (*h_0_* = no channel leads, *α =* 0.05, two-sided, cluster-based permutation with 10,000 resamples). See Supplementary Table 23 for *p*-values. **(B)** Network diagrams illustrating sequence practice-related *δ*-*β^δ^* effects. **(C)** Task exposure-related effects in interregional *δ*-*β^δ^* coherence. Visualization and statistics identical to **(A)** (see Supplementary Tables 24, 25 for *p*-values). **(D)** Network diagrams illustrating task exposure-related *δ*-*β^δ^* effects. GP, globus pallidus; Put, putamen; vSTN, ventral subthalamic nucleus; dSTN, dorsal subthalamic nucleus; M1, primary motor cortex; PM, premotor cortex; S1, primary somatosensory cortex; Par, parietal cortex.

Likewise, there was no evidence for consistent interregional *β* gating of M1 excitability; however, striatocortical *δ* → *β^δ^* coupling developed with sequence learning in GP1 (Figures 8A,B). This contrasts with sequence practice-related corticostriatal *δ*-*β^δ^* coupling in GP2. With task exposure, primary motor cortex led PM in both GP subjects, but only in GP1 was GP led by all recorded brain regions (Put, M1/S1, PM) (Figures 8C,D). In STN subjects, no sequence practice-related effects were observed. However, with task exposure in STN1, Par led M1 and dSTN—effects absent in STN2. These results associate sequence learning and task exposure with unique, anatomically-specific patterns of *δ*-*β* coupling in improvers.

## Discussion

**Figure 9:**
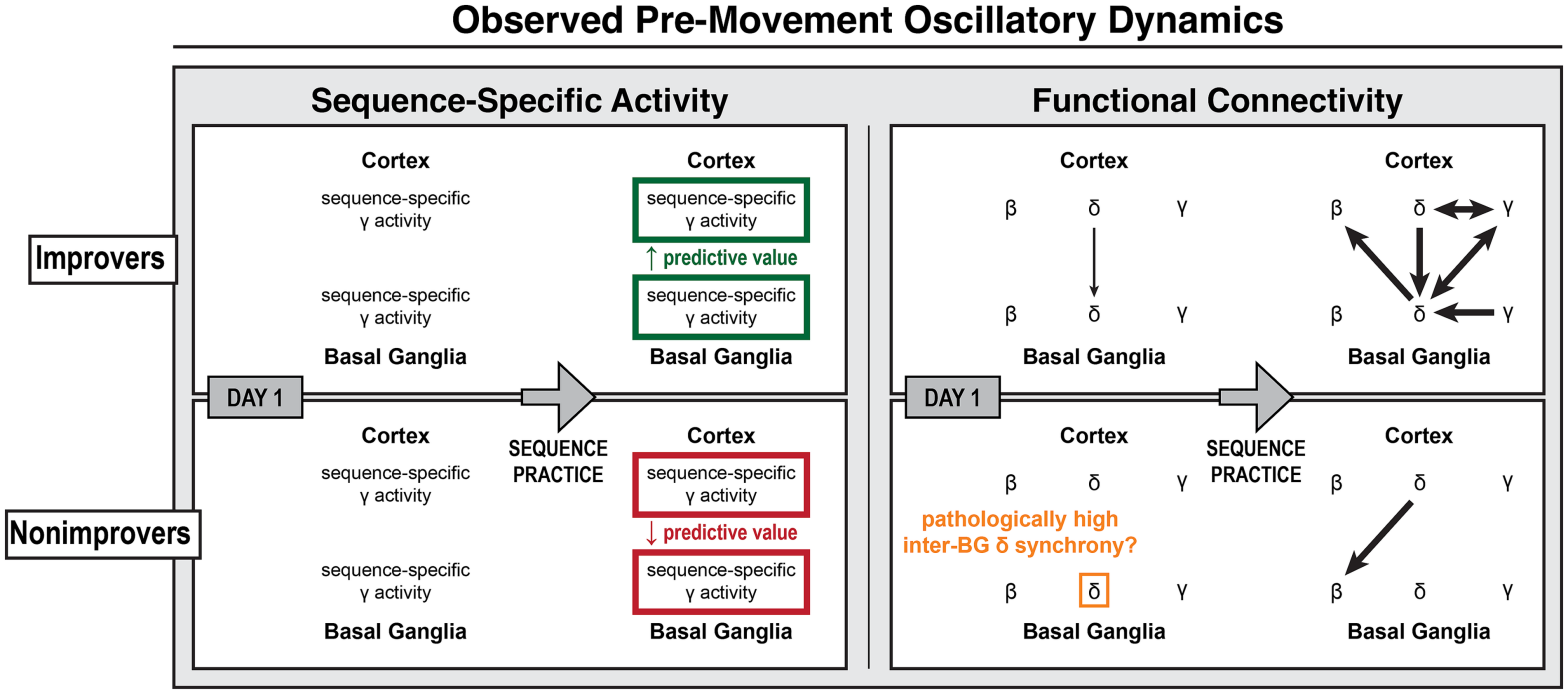
Observed learning-related cortico-basal ganglia activity during motor sequence initiation. Diagram of observed learning-related changes in sequence-specific activity and functional connectivity prior to the onset of motor sequences over multiple days of practice. (Left) Sequence-specific *γ* activity was present in all brain regions for all subjects. With practice, *γ*’s predictive value increased in improvers but decreased in nonimprovers. (Right) As predicted, successful sequence learning involved cortico-basal ganglia *δ* synchrony and *δ* → *γ* coupling with motor cortex *γ*, though *δ* → *γ* coupling was not observed with basal ganglia *γ*. Sequence learning also involved other cross-frequency couplings, most notably *δ* → *γ* and *δ* → *β* couplings in which basal ganglia led cortex. In nonimprovers, highly coherent inter-basal ganglia *δ* was uncoupled from cortex and unresponsive to task events. These results suggest (1) that sequence learning harnessed cortically-led *δ* phase coordination to organize distributed higher-frequency neural activity for the optimization of multi-element preplanning and (2) that pathological BG *δ* synchrony may interfere with BG *δ* phase coding by reducing BG sensitivity to cortical input, ultimately disrupting motor learning.

**Figure 10:**
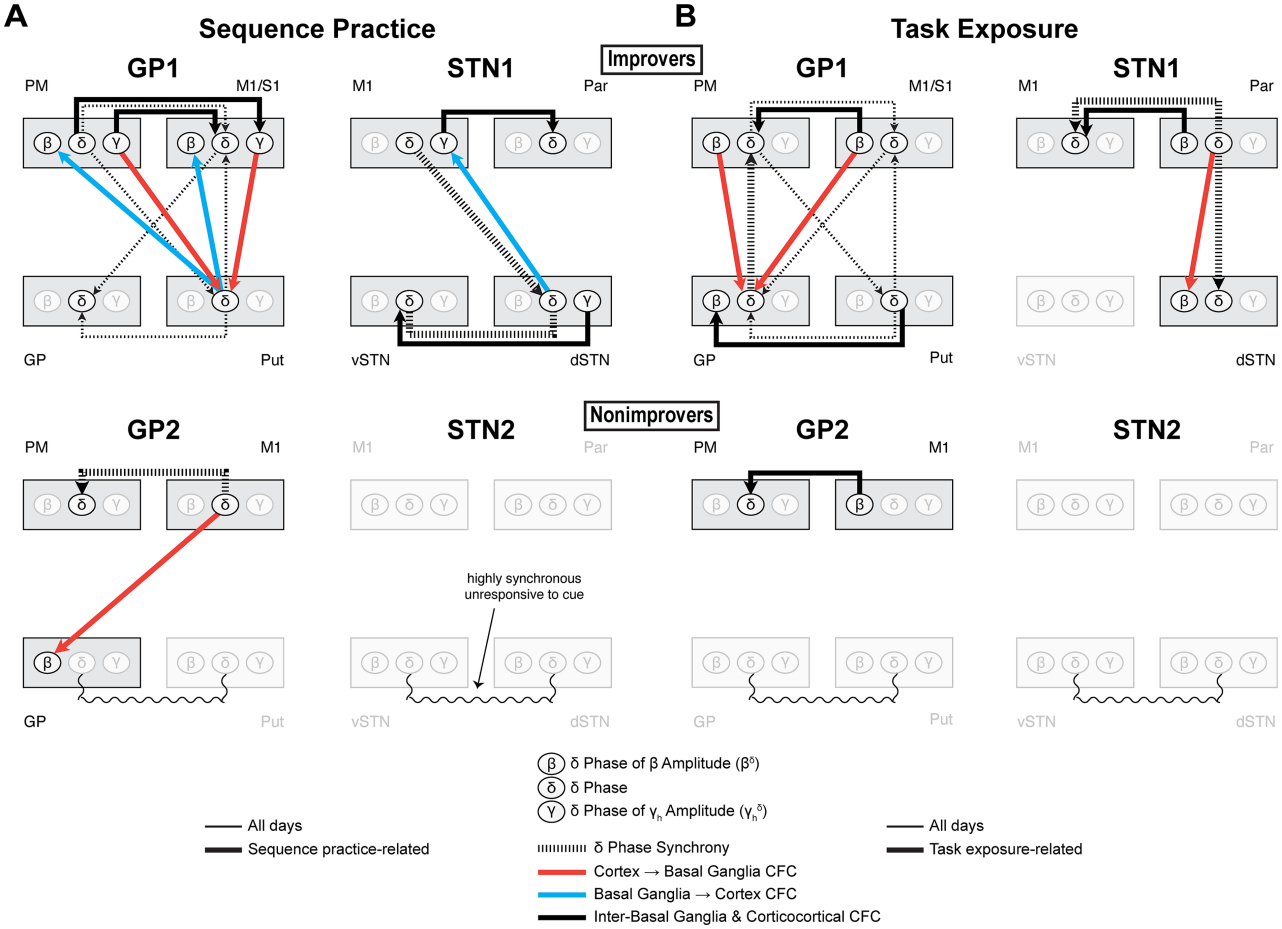
Coordinated *δ* activity supports a hierarchical functional network during sequence practice and task exposure that reflects the specialized roles of cortico-basal ganglia regions. Composite diagrams of interregional effects associated with **(A)** sequence practice and **(B)** task exposure. As motor sequence learning overlaps with increasing familiarity with the experimental process, we dissociated the functional connectivity effects related to sequence practice from those related to task exposure. In improvers, sequence learning and task exposure produced distinct patterns of CFC and changes to network *δ* dynamics, though sequence learning and task exposure were nonetheless linked by a common framework of cortically-led network *δ* synchrony. For each type of communication, the presence and direction of coupling depended on the involved brain regions, indicative of their specialized roles. In nonimprovers, the absence of both sequence practice- and task exposure-related effects involving subcortical *δ* further suggests that a locus of their interdependence may be cortico-basal ganglia *δ* synchrony. Furthermore, in all subjects, only the *δ* in regions that aligned to cue participated in interregional CFC. Except for motor cortex *δ*, this local *δ* alignment to cue was coupled with network *δ* synchrony. These findings support a model wherein a network capable of learning leverages preparatory coordinated network *δ* activity to organize both sequence learning- and task exposure-related effects, with the two effect types distinguished by a unique interplay of *δ* synchrony and CFC. This ultimately poses coordinated network *δ* as an ideal substrate for task familiarization to boost future sequence learning. Neurostimulation that enhances event-related cortico-basal ganglia *δ* coupling may thus prove a broadly effective strategy to improve skilled fine motor control in PD. GP, globus pallidus; Put, putamen; vSTN, ventral subthalamic nucleus; dSTN, dorsal subthalamic nucleus; M1, primary motor cortex; PM, premotor cortex; S1, primary somatosensory cortex; Par, parietal cortex.

This study is the first invasive electrophysiological investigation of human cortico-basal ganglia dynamics in motor sequence learning. Though all subjects demonstrated M1 *β* desynchronization, *δ* phase alignment to cue and *γ* synchronization, a consistent *β* → *δ* → *γ* cascade was absent—possibly reflecting PD-related neuropathophysiology (Figures 9, 10). Instead, *δ*-*β* and *δ*-*γ* coupling emerged with learning, possibly supporting optimization of preparatory activity. However, consistent with our predictions, we observed increasingly predictive sequence-specific cortical and basal ganglia *γ* activity alongside cue-aligned cortico-BG *δ* synchrony in improvers. Strikingly, all brain regions were eventually recruited, producing network *δ* synchrony. Furthermore, M1 *γ*’s increasing predictive value was linked to its coupling with synchronized network *δ*, tying coordinated network *δ* to enhanced recruitment of sequence-specific neural activity. In contrast, in nonimprovers, decreasingly predictive *γ* corresponded with minimal CFC and absent coordinated network *δ* activity. Highly coherent inter-BG *δ* did not synchronize with cortex or align to visual cues, suggesting it was pathological. These results suggest that both cortex and basal ganglia supported multi-element preplanning and that cortico-basal ganglia communication was critical to learning-driven optimization of motor preparation. This highlights how motor learning can harness a hierarchical functional network architecture to optimize information transfer and temporally structure neural activity in PD.

### The *β* → *δ* → *γ* framework for motor initiation: an assessment in Parkinson’s disease

The expected cascade of network *β* → cortical *δ* → cortical *γ* for general motor initiation was absent in our subjects, indicating it was not prerequisite (Ganguly et al., 2022; Fasano et al., 2022; Singh, 2018; Kaufman et al., 2014; Churchland and Shenoy, 2024; Crone et al., 1998; Herrojo Ruiz et al., 2014; Herrojo Ruiz et al., 2014; Jenkinson et al., 2013; Muralidharan and Aron, 2021; Torrecillos et al., 2018; Yin et al., 2021; Pascual-Leone et al., 1994). Our observation of both lead directions for *δ*-*γ* and *δ*-*β* couplings is novel, as most studies have employed undirected coupling measures (Combrisson et al., 2017; Attaheri et al., 2022; Natraj et al., 2022; Ramanathan et al., 2018). Coupling in which *β* or *γ* led *δ* was often associated with enhanced *δ* alignment to cue or lead of other brain regions, suggesting a practice-driven role for *β*- and *γ*-related neural activity in influencing network *δ* dynamics. This may reflect compensatory or pathological mechanisms related to increased cortical *δ* sometimes observed with PD progression (Pal et al., 2020; Ponsen et al., 2013; Rucco et al., 2022).

### Practice-driven pre-movement sequence-specific activity

Pre-movement sequence-specific activity has been observed in nonhuman primate motor cortex, and we provide the first electrophysiological evidence of it in human cortex and basal ganglia (Lu and Ashe, 2005; Hatsopoulos et al., 2003). Among canonical frequency bands, *γ*’s large bandwidth necessitated the most narrowband filters for time-frequency analysis, which inherently increased its representation in the total feature set. However, lasso regularization and within-time-frequency percentile-based feature selection helped prioritize features based on sparsity and discriminative value. Thus, sequence-specific *γ* activity may have arisen from unique neural ensembles, with distinct firing patterns and spatial proximities to the recording contacts resulting in differing field potential dynamics (Chang, 2015; Ray et al., 2008).

While animal model motor learning studies suggest that increasingly predictive cortical and BG *γ* should couple with network *δ*, we did not observe *δ* → *γ* for BG *γ* (Khanna et al., 2021; Ganguly and Carmena, 2009; Peters et al., 2014; Rostami et al., 2024; Guo et al., 2021; Ganguly et al., 2022; Lemke et al., 2019). Unrecorded regions may have facilitated BG *γ*’s increased predictive value. Alternatively, *δ* may have indirectly influenced sequence-specific activity through its lead of *β*, which may bind action plan-specific ensembles across the network (Ganguly et al., 2022). Decreased decoding accuracy in some brain regions of nonimprovers may have reflected reduced consistency of ensemble activity, potentially contributing to lack of motor improvement. Given our small sample size and recordings at the level of neural populations rather than individual neurons, these suggestions are conjecture and should inspire future experimental work.

### Sequence learning and task exposure produced distinct patterns of cortico-basal ganglia functional connectivity in improvers

Different patterns of both CFC and *δ* synchrony emerged with sequence learning and task exposure in improvers. For CFC, most notably, basal ganglia→cortex CFC developed only with sequence learning, with putamen and dSTN *δ* leading *β* and *γ* in cortex. This aligns with models implicating putamen and dSTN in action selection and learning and suggests that they perform these roles through CFC patterning of high-frequency cortical activity (Doyon, 2008; Marinelli et al., 2017; Rusu and Pennartz, 2020; Caligiore et al., 2019; Simonyan, 2019; Klaus et al., 2019; Balleine et al., 2015; Milardi et al., 2019). For *δ* synchrony, consistent with neuroimaging, we found corticostriatal *δ* coherence in GP1 (Toni et al., 2002; Doyon et al., 2003; Debas et al., 2014). We further established a cortex→striatum direction of lead and found sequence learning-related M1 → dSTN connectivity. In contrast, task exposure drove striatocortical *δ* synchrony. GP → PM *δ* synchrony completed a possible loop of cortico-basal ganglia *δ* phase coordination, via PM → Put→GP → PM. This suggests that general motor familiarization or the optimization of attentional processes could involve a positive feedback loop of network *δ* synchrony, which could aid future sequence learning (Combrisson et al., 2017; Canavier, 2015; Harmony, 2013; Voloh and Womelsdorf, 2016).

### Few effects of sequence practice and task exposure in nonimprovers: possible impact of pathologically synchronized basal ganglia *δ*

The absence of cue-related functional connectivity involving BG *δ* in nonimprovers may have been related to high session-wide inter-BG *δ* synchrony. Human and animal studies have detected *δ*-range spiking activity in GP and STN in low dopamine states and linked it to motor symptoms, suggesting that ineffective or absent dopamine replacement therapy can permit pathological BG *δ* activity (Levy et al., 2002; Zhuang et al., 2019; Whalen et al., 2020). Consistent with this, our subjects exhibiting elevated BG *δ* synchrony were those for whom general motor function remained the most compromised while on dopamine medication (Supplementary Table 1). However, their daily post-task UPDRS upper limb scores were comparable to those of other subjects and demonstrated no apparent across-day drop alongside the across-day decrease in BG *δ* synchrony (Supplementary Table 3). It is possible that task exposure drove the across-day BG *δ* synchrony decrease, promoting the possibility of future learning, and that the MDS-UPDRS upper limb component was too insensitive to detect associated changes in hallmark upper limb motor symptoms (Tosin et al., 2021). In any case, the concurrent lack of improvement and absence of practice-related effects involving BG *δ* suggests that event-related coordination of BG *δ* may have been important to motor learning in these subjects.

### Coordinated network *δ* as a facilitatory network state for learning-dependent cross-frequency coupling during sequence initiation

From the theoretical standpoint, *δ* is an ideal substrate for information multiplexing, local gain modulation and information transfer between distant phase-aligned brain regions (Hahn et al., 2019; Schroeder and Lakatos, 2009; Canavier, 2015; Harmony, 2013; Voloh and Womelsdorf, 2016). Experimental evidence supports this, showing a role for *δ* in information sequencing through phase-specific ensemble patterning; sensory integration and attentional control through gain modulation achieved by changes in neural excitability; and functional network organization, information transfer and distributed representation through cross-area coordinated activity that coactivates distributed ensembles (Khanna et al., 2021; Hahn et al., 2019; Schroeder and Lakatos, 2009; Lakatos et al., 2008; Whittingstall and Logothetis, 2009; Lemke et al., 2019; Natraj et al., 2022; Dann et al., 2016; Wyart et al., 2012).

Consistent with these roles, in our study, cross-area coordination of *δ* phase may have formed the infrastructure for a cue-responsive network state, within which learning-related *δ*-*β* and *δ*-*γ* coupling developed. While *δ* synchrony and phase alignment to cue occurred on Day 1 and increased across days, interregional *δ*-*β* and *δ*-*γ* directed couplings were initially mostly absent. Moreover, only the *δ* in brain regions that aligned to cue participated in interregional CFC, and, outside of motor cortex, local *δ* alignment to cue was always linked to network *δ* synchrony. These results highlight a potential role for cross-area *δ* synchrony in driving consistent event-related local *δ* phase dynamics, which can, in turn, support phase coding that patterns high-frequency activity.

This link between motor performance-related *δ* activity and CFC is consistent with lesion experiments in animal models and observational studies of the human cortical grasp network (Khanna et al., 2021; Lemke et al., 2019; Natraj et al., 2022). Other work has found reaction time-correlated cue-responsive *δ* phase reset in human hippocampus; reaction time-correlated *δ* phase in rat motor thalamus coupled to local spiking; motor learning-related M1-cerebellar *δ* synchrony in rats with enhanced cross-area spiking activity and even evidence for prefrontal guidance of motor plans via *δ*-*β* coupling in humans (Riddle et al., 2021; Kleen et al., 2016; Gaidica et al., 2020; Fleischer et al., 2023). In humans with PD, increased prefrontal *δ* activity during sleep with low frequency stimulation of the basal ganglia may improve memory retention, suggesting importance of this signal to offline learning as well (Herz et al., 2025). Coordinated *δ* may ultimately reflect a system-wide neural process facilitating local and distributed activity patterns over the course of learning.

However, the neurophysiological basis of the activity reflected in *δ* remains unclear. While *δ* has been associated with excitability and with single-unit spiking at *δ* frequency, it has also, similar to *γ*, been linked to the dynamics of population spiking activity (Ramanathan et al., 2018; Hall et al., 2014), and it could reflect synaptic input (Haberly and Shepherd, 1973; Rebert, 1973; Mitzdorf, 1985; Parker et al., 2002). It is thus important to clarify that *δ* activity may not directly influence other neural activity, per sé, but rather reflect the underlying activity that drives the observed network effects.

### Clinical implications

Parkinson’s disease often involves an impairment of action sequencing, but its neural basis is poorly understood and incompletely remediated by dopamine replacement therapy and conventional DBS (Marinelli et al., 2017; Park, 2017; Heilman, 2020). Our observation that both sequence learning and task exposure involved cortico-basal ganglia *δ* phase coordination in improvers suggests that enhancing event-related cortico-basal ganglia *δ* synchrony may improve not only the learning of specific sequences but also the ability to adapt to new cued motor tasks. Improved task learning could, in turn, boost future sequence learning for a range of sequences or contexts, suggesting therapies aimed at restoring cortico-basal ganglia *δ* synchrony could be broadly effective for skilled hand movements. Electrically stimulating basal ganglia upon detection of motor intention and based on the ongoing phase of BG or cortical *δ* could promote cortico-basal ganglia *δ* synchrony and possibly disrupt pathological BG *δ*. Thus, our findings posit pre-movement *δ* phase-specific striatal or subthalamic DBS as a therapeutic neuromodulatory strategy to restore fine motor control.

### Central limitations

Our study was limited by a small sample size, as is common in human invasive electrophysiology. Anatomically nonoverlapping recordings in GP and STN subjects revealed the specialized neural activity of different brain regions but left unverifiable the consistency of effects across improvers or across nonimprovers. The lack of improvement in half the subjects allowed binary behavioral stratification but limited analysis to within-subjects comparisons with *n* = 1 per learner type per brain region. Future work should replicate this study in larger cohorts.

It is possible that performance floor effects led to incorrect behavioral stratification. Despite lack of performance improvement, nonimprovers typed at least as quickly as improvers. This indicates that lack of improvement was not due to difficulty manipulating the keyboard and could suggest that nonimprovers found the task too easy at baseline to show measurable improvement with sequence learning. However, performance decrements when switching between sequences within a given day—indicative of interference caused by sequence learning—were not apparent in nonimprovers’ single-trial performance data. It is possible that initial typing speeds reflected typical inter-individual variability.

## Conclusion

In individuals experiencing Parkinson’s disease, we outline a hierarchical, learning-dependent functional architecture of oscillatory cortico-basal ganglia activity for the initiation of fine motor sequences. The findings illuminate how disparities in information content and flow may relate to disparities in motor learning outcomes. Extending this work in larger cohorts of individuals with PD could help elucidate the relationship between clinical characteristics and practice-related neural dynamics. This would further clarify the potential for phase-specific basal ganglia stimulation to modulate pathological neural dynamics in the production of fine motor skills.

## Data Availability

The dataset presented in this article is not readily available because we are legally bound by our contracts with UCSF and Medtronic not to publicly share patient data. For such a small cohort of patients, there are also privacy concerns. Requests to access the datasets should be directed to doris.wang@ucsf.edu.
