# Supplementary material for: Motor learning leverages coordinated low-frequency cortico-basal ganglia activity to optimize motor preparation in humans with Parkinson’s disease
Source: Front Neurosci. 2025 May 13;19:1542493. doi: 10.3389/fnins.2025.1542493 (PMC12106502; doi:10.3389/fnins.2025.1542493)
Supplement: Supplementary file 1 [file Data_Sheet_1.PDF]

SUPPLEMENTARY MATERIALS: FIGURES AND TABLES

SUPPLEMENTARY FIGURES

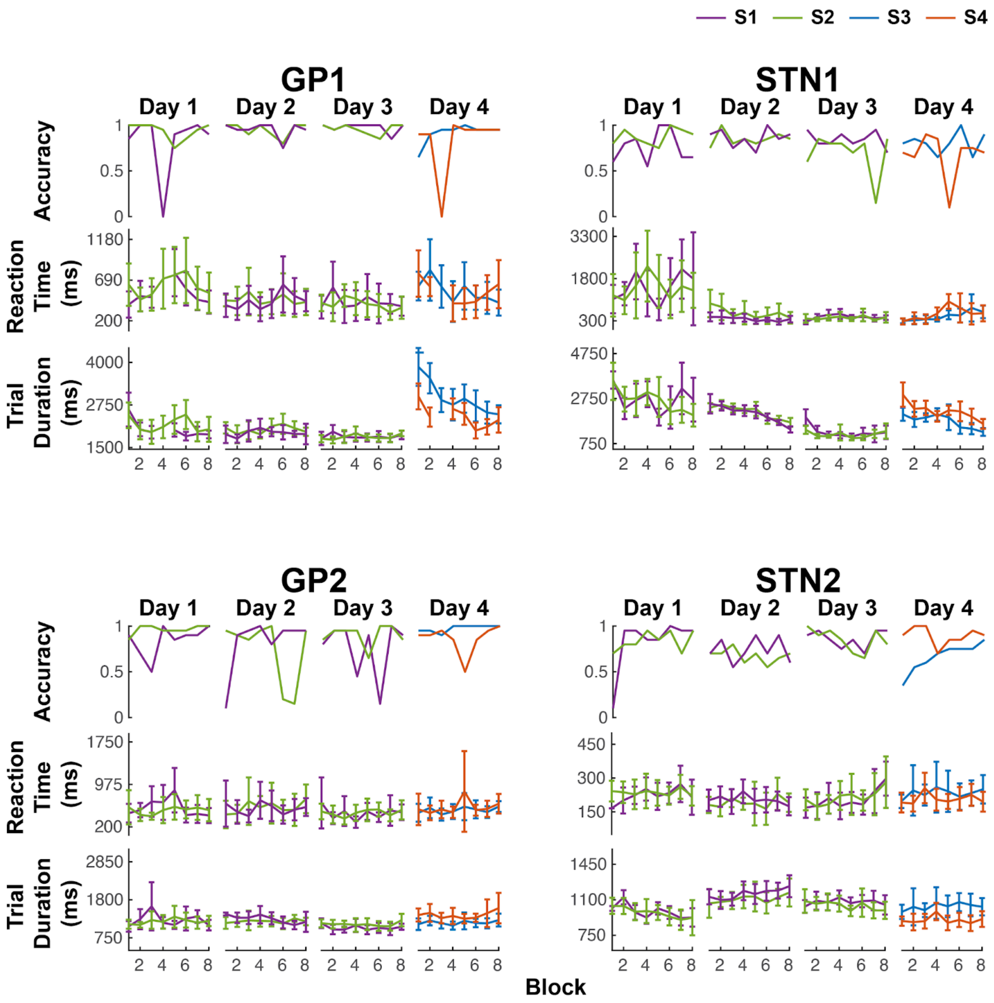

Supplementary Figure 1. Block average performance data.

Per subject: (Top) block average accuracy, (Middle) block average reaction time [cue onset to movement onset], (Bottom) block average trial duration [movement onset to offset]. Error bars indicate ± *s*.

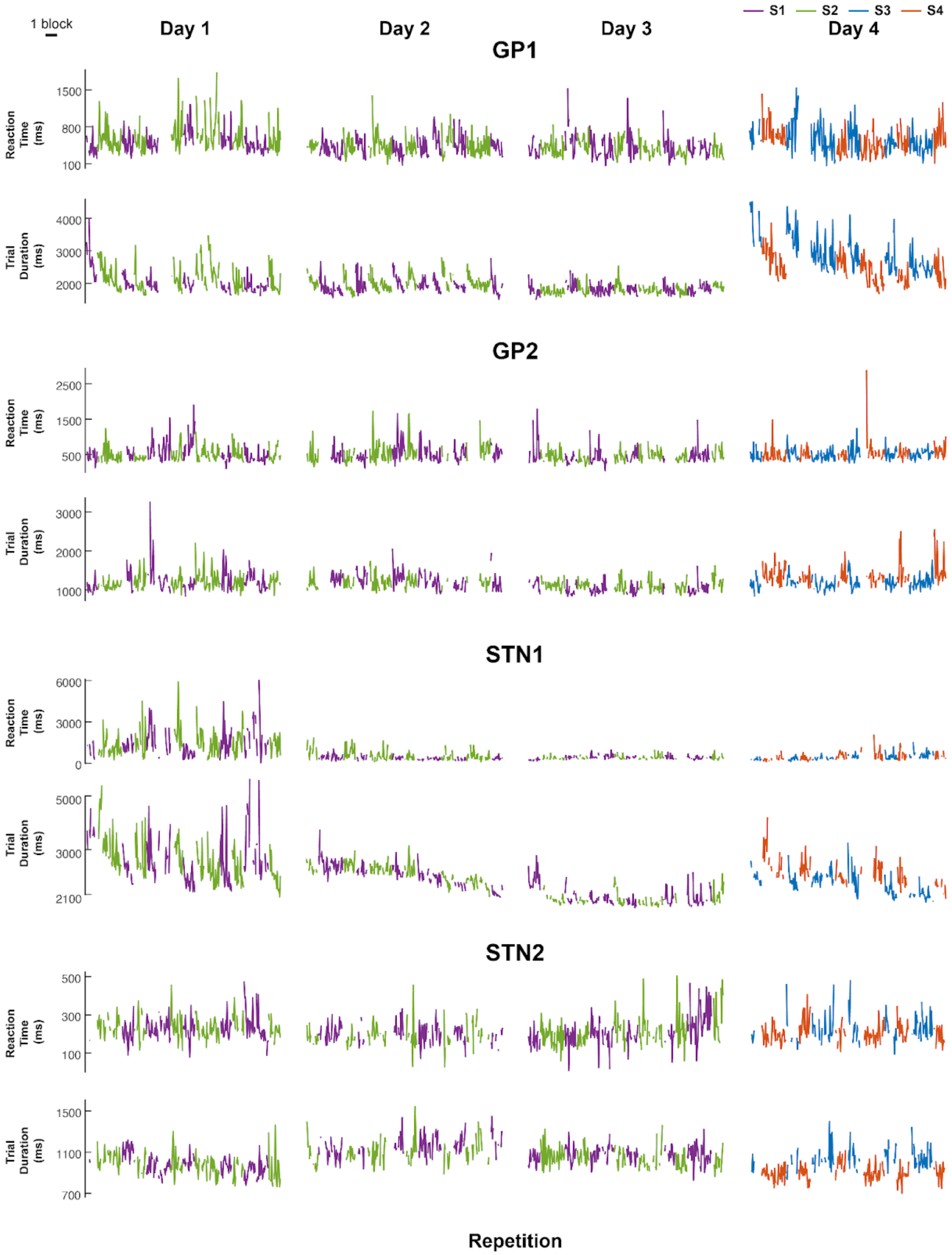

Supplementary Figure 2. Single-trial performance data.
Single-trial reaction time and trial duration for all fully correct trials.

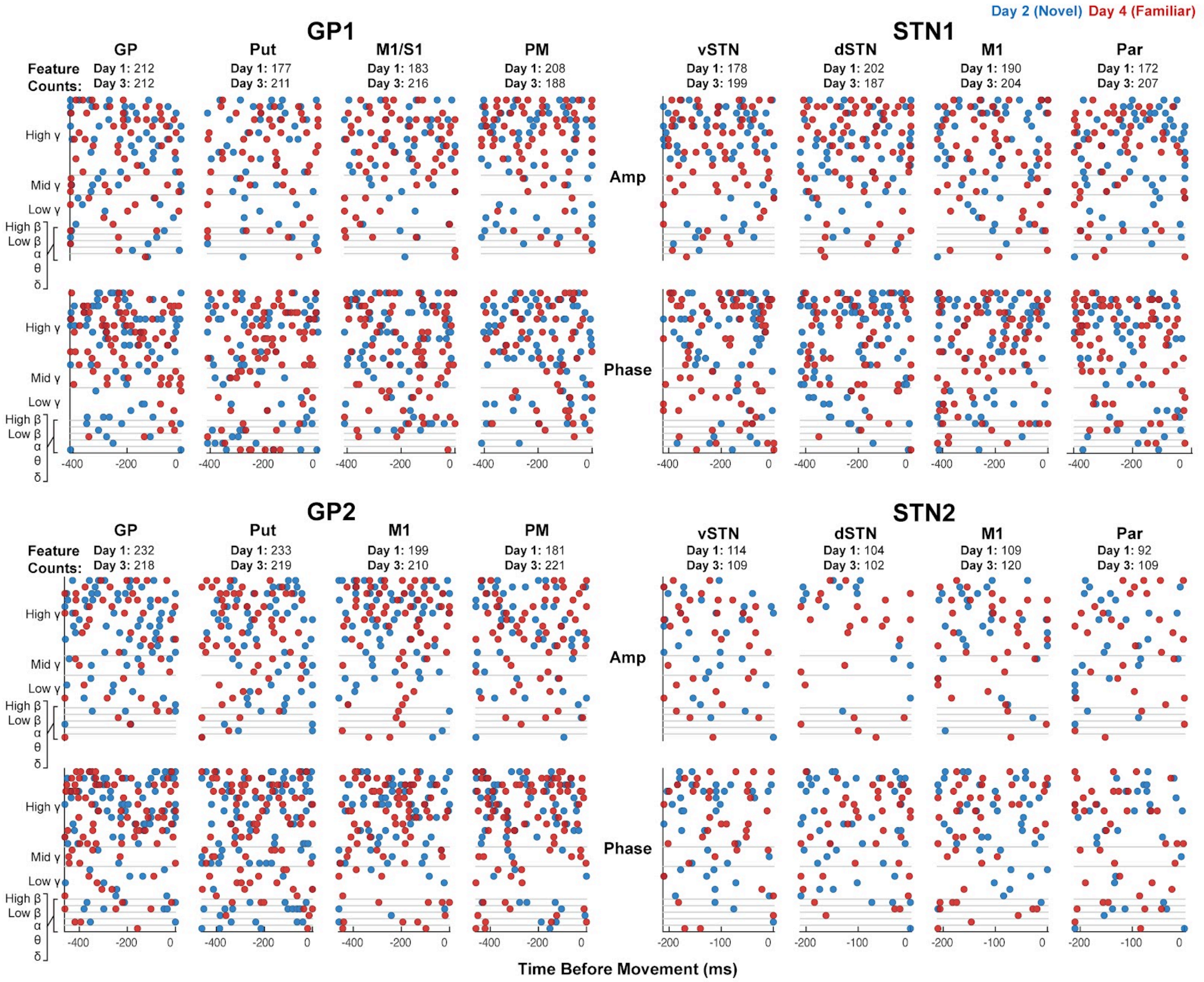

Supplementary Figure 3. Features selected prior to lasso regularization.
Features were selected separately for each model. Total feature count per model (i.e., per subject per channel per day) prior to lasso regularization is shown above each channel's amplitude and phase feature plots. ***Feature counts are based on each design matrix and thus include a feature for both the sin(phase) and the cos(phase) of each selected phase time-frequency point.*** As a larger number of phase than amplitude time-frequency points tended to reach the 80th percentile cutoff for feature selection, the indicated total feature counts are at least 50% higher than the number of initially selected time-frequency points.

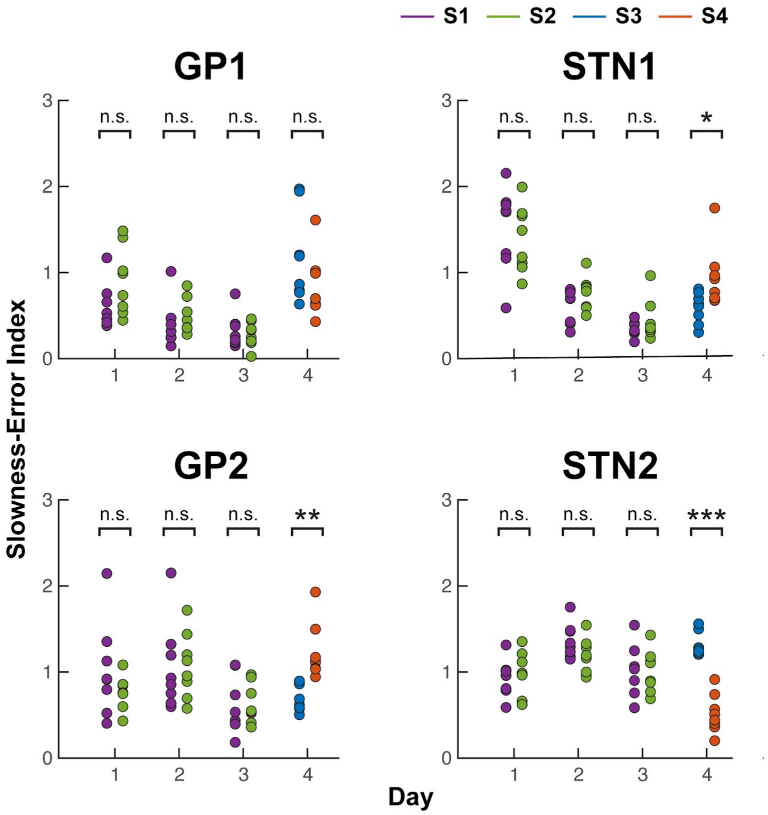

Supplementary Figure 4. On Days 1–3, overall sequence performance was comparable between S1 and S2.

Across-sequence comparison of performance within each day assessed differences in overall performance level ($\alpha = 0.05$, two-sided, two-sample t-test with unequal variance, $n = 8$ sequence blocks per group except for GP1 Day 1 S1 for which $n = 7$ due to exclusion of 0% accuracy blocks, as composite performance would be poorly defined.). $^{*}p < 0.05$, $^{**}p < 0.01$, $^{***}p < 0.001$.

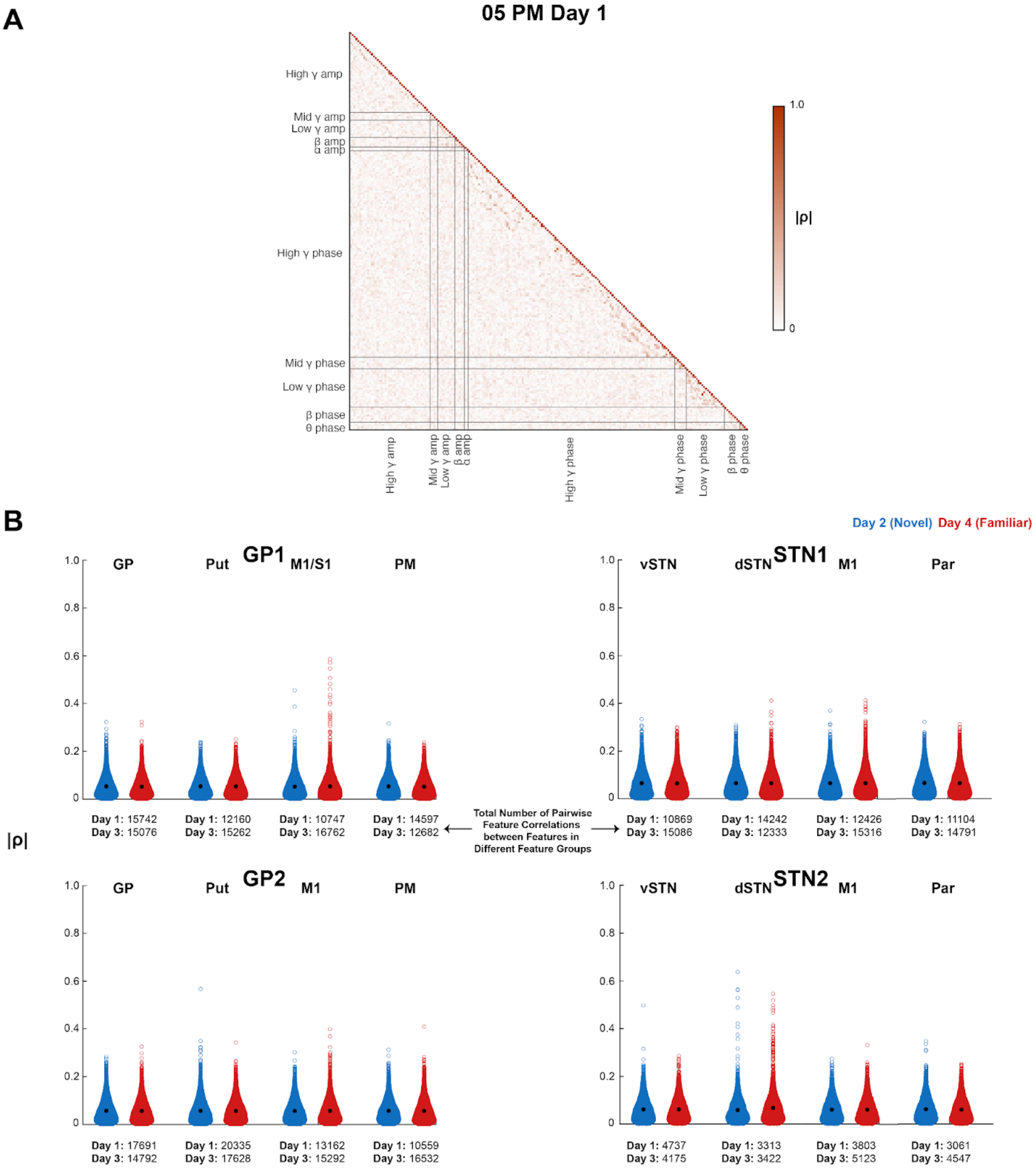

Supplementary Figure 5. Correlations between features after grouping by canonical frequency band and signal property.
(**A**) Example feature correlation matrix for a single model, where light gray lines separate feature groups. (**B**) For each model, a swarm plot of all possible feature correlations between features in different groups. Features were grouped by canonical frequency band and also by signal property, i.e., δ phase features were grouped separately from δ amplitude features, as well as from all other canonical frequency bands. Total number of feature correlations between features in different feature groups for a given model is shown below corresponding swarm plots.

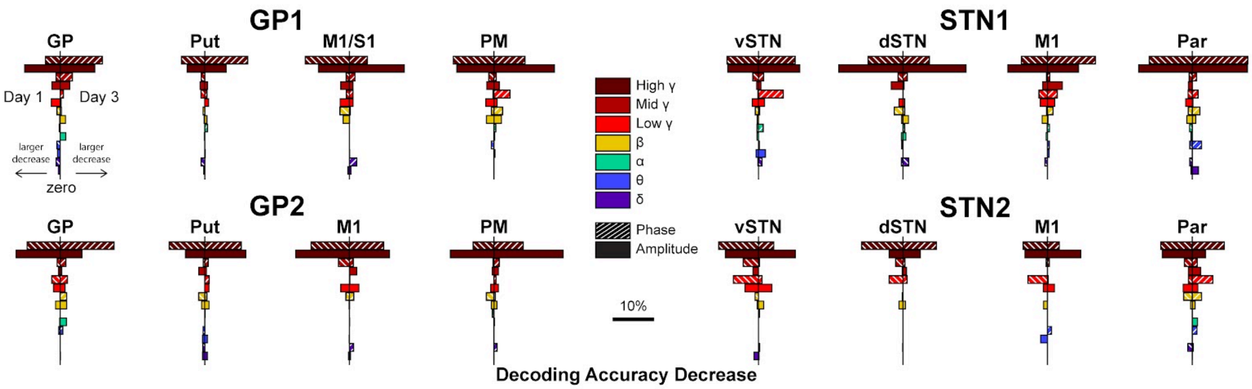

Supplementary Figure 6. Absolute decoding accuracy decreases for features grouped by canonical frequency band and signal property.

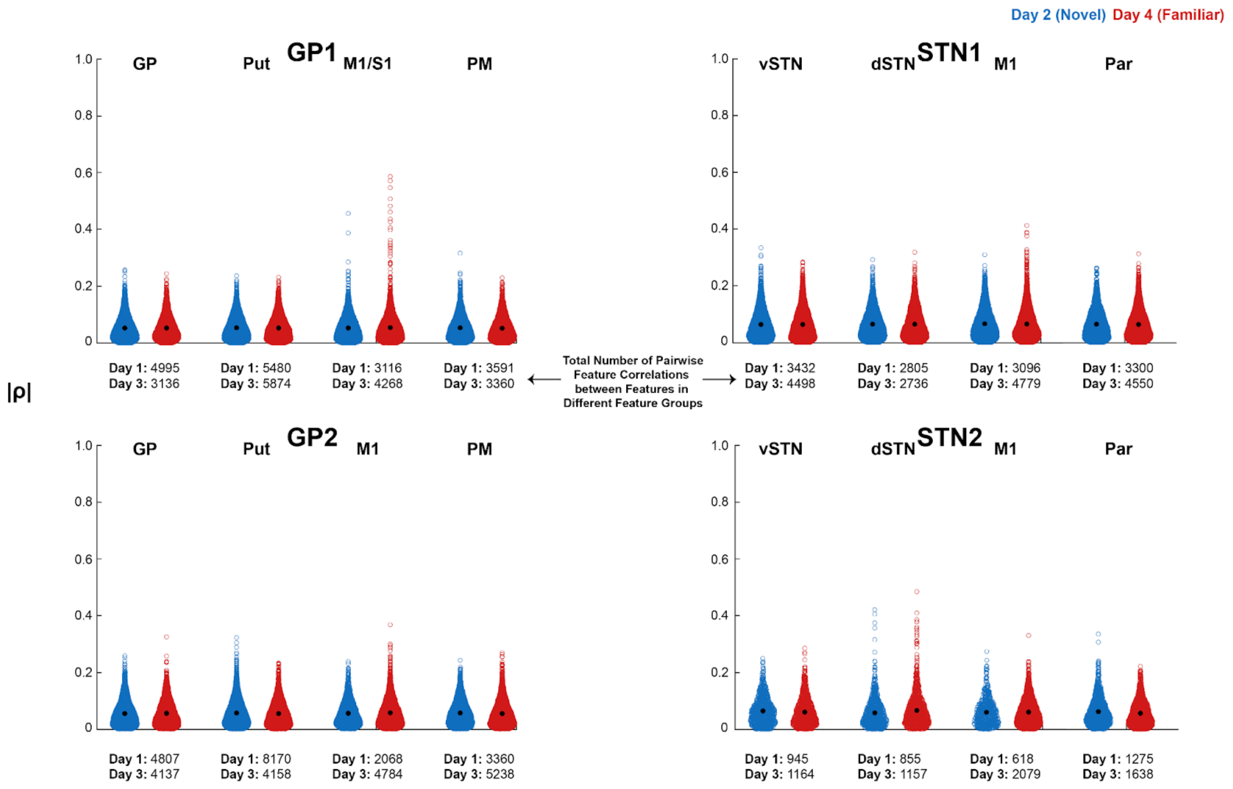

Supplementary Figure 7. Correlations between features after grouping into 1) δ through β and 2) low γ through high γ.

For each model, a swarm plot of all possible feature correlations between features in different groups. Features were grouped into δ through β (0.5–30 Hz) and low γ through high γ (30–250 Hz), with amplitude and phase features grouped together. Total number of feature correlations between features in different groups for a given model is shown below corresponding swarm plots.

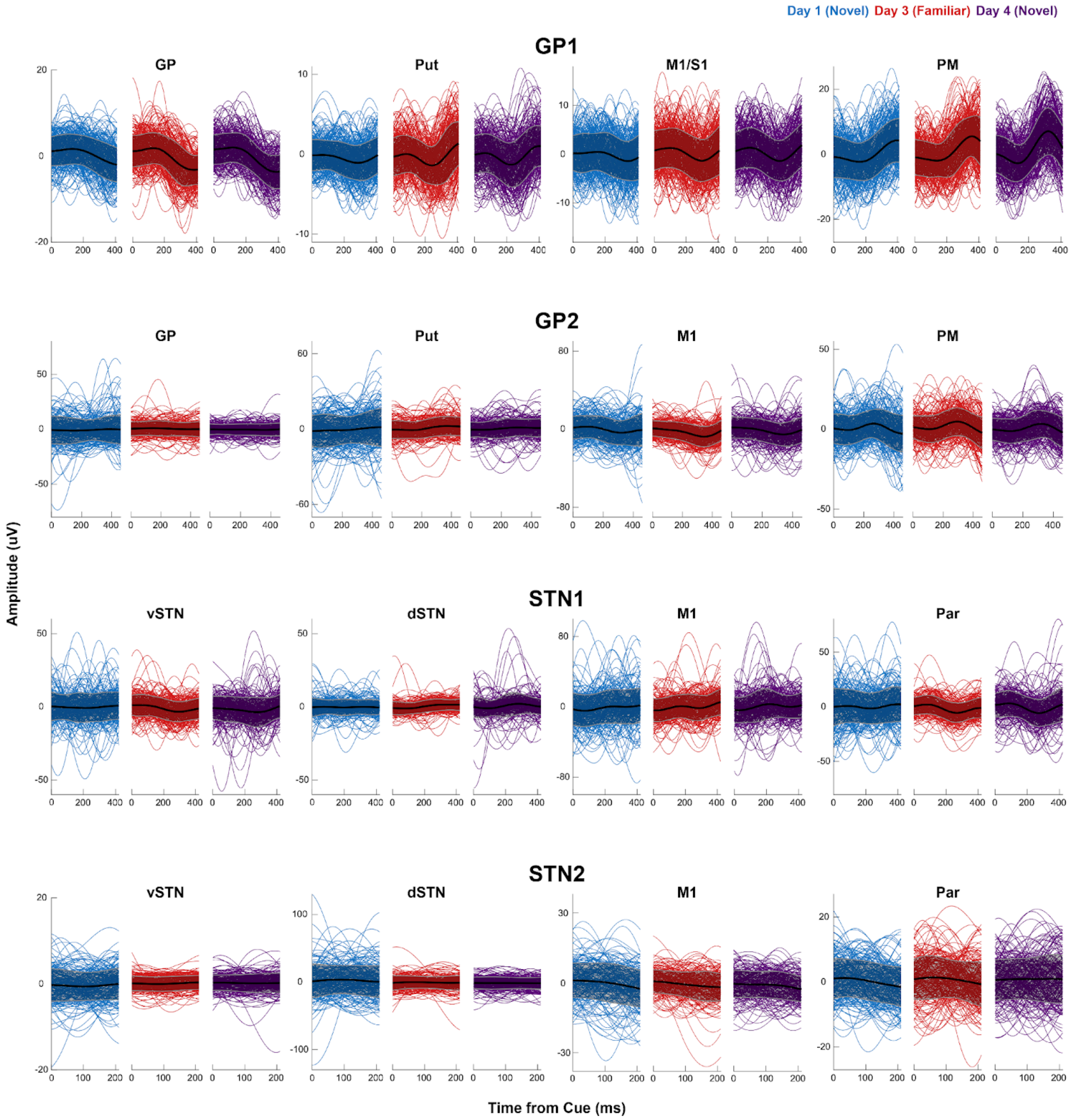

Supplementary Figure 8. Single-trial δ time domain data.
Data is aligned to cue onset and plotted as $\bar{x} \pm s$.

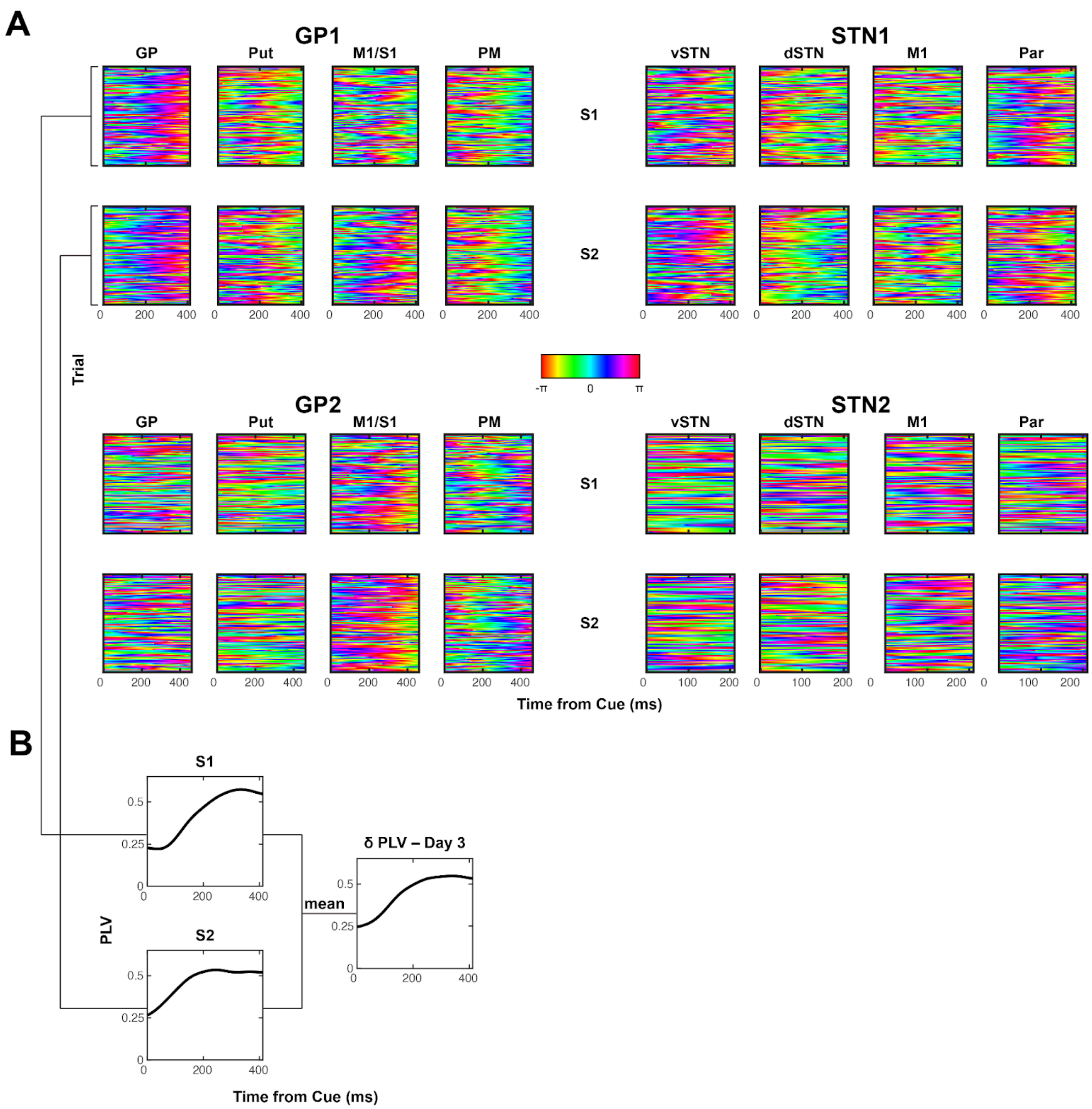

Supplementary Figure 9. Day 3 single-trial δ phase.
(**A**) δ phase data aligned to cue onset. (**B**) Example of phase locking value calculation. Phase locking value was first calculated within sequence and smoothed with a 150 ms-long Gaussian window. Resulting time series were averaged across the two sequences to compute the overall phase locking value for this brain region on this day.

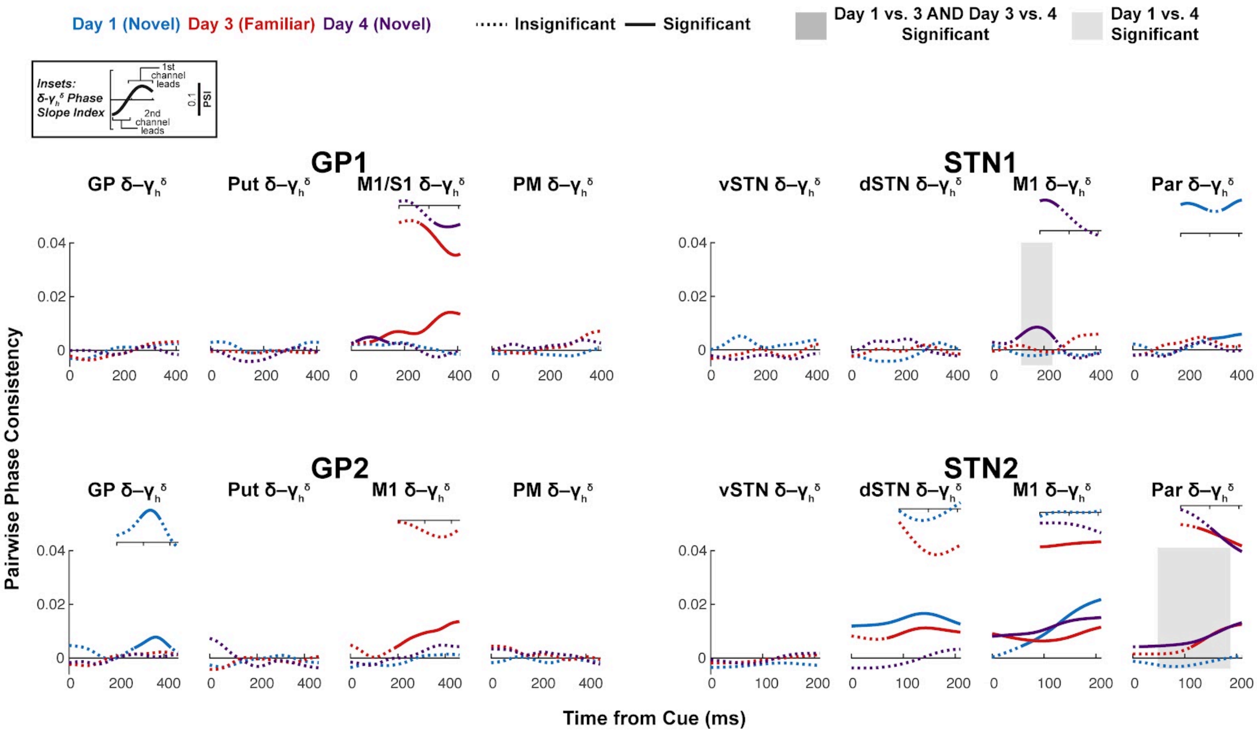

Supplementary Figure 10. Intraregional δ-γ coupling.

(Large plots) Pairwise phase consistency (PPC, undirected measure) was calculated between δ phase and the δ phase of the high γ amplitude envelope. Solid line indicates significant PPC (h_0 = coherence is not higher than expected given the phase distribution, α = 0.05, one-sided, cluster-based permutation with 10,000 resamples. See **Supplementary Table 10** for *p*-values.). Shaded box indicates significant difference in PPC between days (α = 0.05, two-sided, cluster-based permutation with 10,000 resamples. See **Supplementary Table 11** for *p*-values.). (Insets) Phase slope index (PSI, directed measure) for data series in which PPC was significant. PSI is displayed for the same total time window as PPC. Solid line indicates significant PSI (h_0 = no channel leads, α = 0.05, two-sided, cluster-based permutation with 10,000 resamples. See **Supplementary Table 12** for *p*-values.).

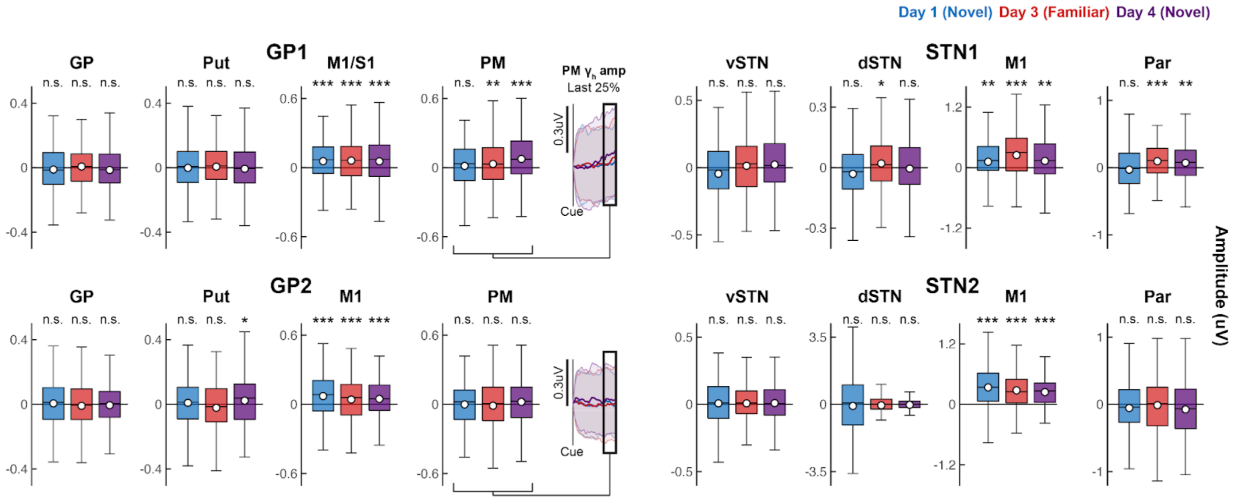

Supplementary Figure 11. High γ amplitude distributions.

(Box plots) Change in high γ amplitude from cue onset to the last 25% of the reaction time period, after linear interpolation of all RT period trials to the same length and smoothing of γ amplitude across time (α = 0.05, one-sided, bootstrap estimation of $\bar{x}$ with 10,000 resamples. See **Supplementary Table 13** for p-values.). White circle reflects mean; black horizontal line reflects median. Box edges correspond to 25th and 75th percentiles. Whiskers span entire data range excluding outliers. Outliers were computed as 1.5·IQR away from the upper or lower quartile and are not shown. (Insets) GP1 and GP2 trial average γ amplitude time series in premotor cortex, with black rectangle indicating the window over which γ amplitude is averaged for individual trials. Error bars indicate ± s. *$p < 0.05$, **$p < 0.01$, ***$p < 0.001$.

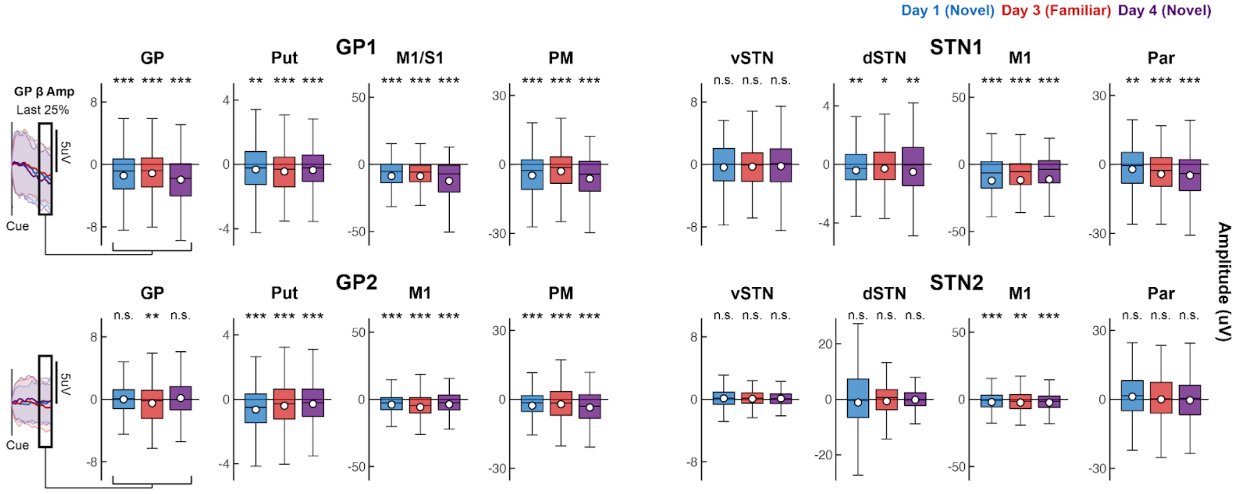

Supplementary Figure 12. β amplitude distributions.
(Box plots) Change in β amplitude from cue onset to the last 25% of the reaction time period, after linear interpolation of all RT period trials to the same length and smoothing of β amplitude across time (α = 0.05, one-sided, bootstrap estimation of $\bar{x}$ with 10,000 resamples. See **Supplementary Table 17** for p-values.). White circle reflects mean; black horizontal line reflects median. Box edges correspond to 25th and 75th percentiles. Whiskers span entire data range excluding outliers. Outliers were computed as 1.5·IQR away from the upper or lower quartile and are not shown. (Insets) GP1 and GP2 trial average β amplitude time series in pallidum, with black rectangle indicating the window over which β amplitude is averaged for individual trials. Error bars indicate ± s. *$p < 0.05$, **$p < 0.01$, ***$p < 0.001$.

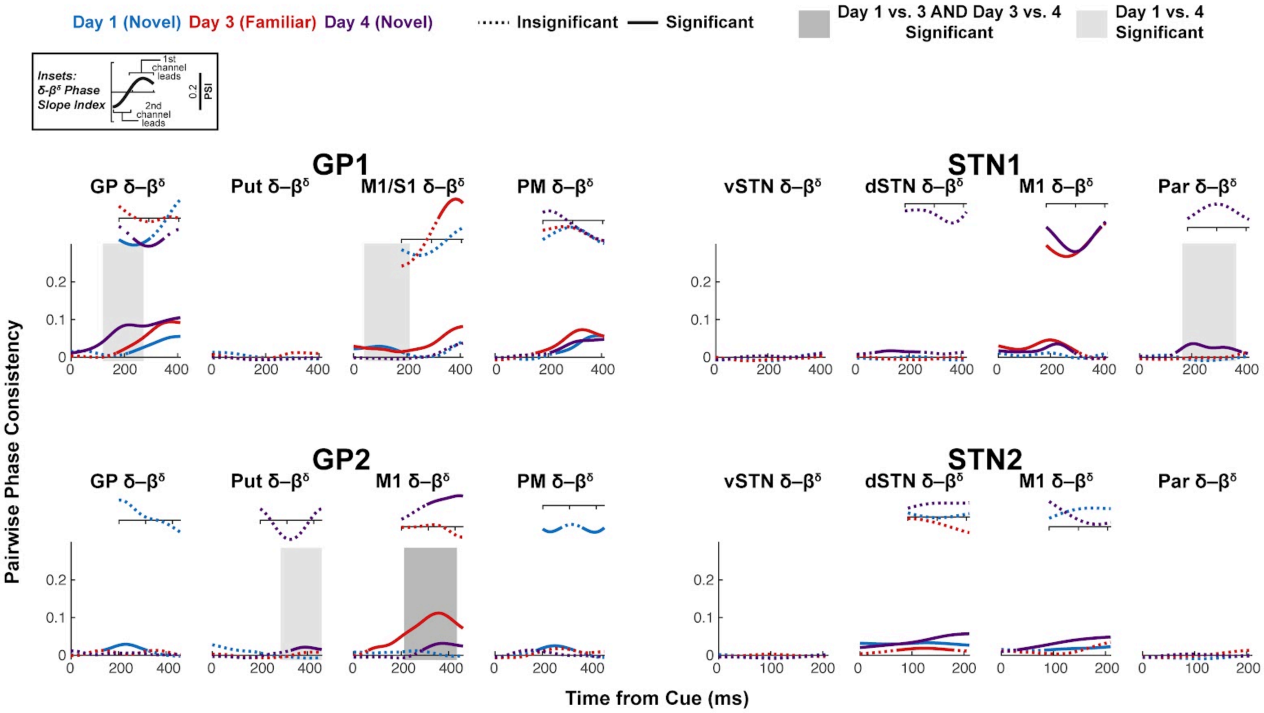

Supplementary Figure 13. Intraregional δ-β coupling.

(Large plots) Pairwise phase consistency (PPC, undirected measure) was calculated between δ phase and the δ phase of the β amplitude envelope. Solid line indicates significant PPC (h_0 = coherence is not higher than that expected given the phase distribution, α = 0.05, one-sided, cluster-based permutation with 10,000 resamples. See **Supplementary Table 18** for *p*-values.). Shaded box indicates significant difference in PPC between days (α = 0.05, two-sided, cluster-based permutation with 10,000 resamples. See **Supplementary Table 19** for *p*-values.). (Insets) Phase slope index (PSI, directed measure) for data series in which PPC was significant. PSI is displayed for the same total time window as PPC. Solid line indicates significant PSI (h_0 = no channel leads, α = 0.05, two-sided, cluster-based permutation with 10,000 resamples. See **Supplementary Table 20** for *p*-values.).

SUPPLEMENTARY TABLES

Supplementary Table 1. Subject demographic and clinical information.
MoCA, Montreal Cognitive Assessment; UPDRS, Unified Parkinson's Disease Rating Scale. *Given the small sample size, age and sex have been omitted to retain participant privacy.*

	Dominant Hand	Pre-Op MoCA	Pre-Op UPDRS ON Med	Pre-Op UPDRS OFF Med	% Change ON Med	Primary Symptoms
**GP1**	R	28	9	18	-50%	Hand tremor, bradykinesia, gait
**GP2**	R	27	17	31	-45%	Hand tremor, bradykinesia, gait
**STN1**	R	26	5	32	-84%	Hand tremor, bradykinesia
**STN2**	R	28	10	31	-68%	Hand tremor, bradykinesia

Supplementary Table 2. Sequences.
No sequences contained repeated adjacent elements, rising or falling triplets, or the thumb. All sequences paired for comparison within and across days started with the same first and last elements. Block order of Sequence 1 and Sequence 2 was switched on Day 2.

	Familiarization	Day 1	Day 2	Day 3	Day 4
**GP1**	2-4-5-3-2<br>2-3-5-4-2	4-2-3-5-4<br>4-5-3-2-4	4-5-3-2-4<br>4-2-3-5-4	4-2-3-5-4<br>4-5-3-2-4	4-2-5-3-4<br>4-3-5-2-4
**GP2**	2-5-3-4-2<br>2-4-3-5-2	3-4-2-5-3<br>3-5-2-4-3	3-5-2-4-3<br>3-4-2-5-3	3-4-2-5-3<br>3-5-2-4-3	3-2-4-5-3<br>3-5-4-2-3
**STN1**	2-4-3-5-2<br>2-5-3-4-2	3-4-2-5-3<br>3-5-2-4-3	3-5-2-4-3<br>3-4-2-5-3	3-4-2-5-3<br>3-5-2-4-3	3-2-4-5-3<br>3-5-4-2-3
**STN2**	2-4-5-3-2<br>2-3-5-4-2	4-2-3-5-4<br>4-5-3-2-4	4-5-3-2-4<br>4-2-3-5-4	4-2-3-5-4<br>4-5-3-2-4	4-2-5-3-4<br>4-3-5-2-4

Supplementary Table 3. Supplementary data collection.
Scores for the upper limb component of the UPDRS performed immediately after the typing task each day and the prior night's sleep duration, collected with a sleep journal. F, Familiarization.

	Day	Postural Tremor	Kinetic Tremor	Finger Tapping	Hand Movements	Pronation/ Supination	Total	Prior Night's Sleep (hr)
**GP1**	**F**	1	1	2	2	1	7	7.75
	**1**	1	0	3	2	3	9	4
	**2**	1	1	3	2	2	9	8
	**3**	2	1	3	2	1	9	7
	**4**	1	1	2	2	2	8	7
**GP2**	**F**	1	2	2	3	1	9	7.5
	**1**	1	1	3	3	2	10	7
	**2**	1	2	2	2	1	8	8.25
	**3**	1	2	2	2	1	8	8
	**4**	2	2	2	2	1	9	8
**STN1**	**F**	1	0	2	1	2	6	7
	**1**	2	1	0	0	1	4	7.25
	**2**	1	0	0	1	0	2	8.5
	**3**	0	0	1	1	1	3	5.25
	**4**	1	0	0	0	1	2	8.75
**STN2**	**F**	1	0	2	2	1	6	6
	**1**	1	0	2	2	1	6	6.5
	**2**	0	1	2	2	1	6	9.5
	**3**	0	1	2	1	1	5	8
	**4**	0	1	2	2	1	6	8.5

(R/task hand)
Range: [0, 4] 0 = none, 4 = severe

Supplementary Table 4. Baseline testing of local cue-related δ phase locking value: *p*-values. Blank cells indicate no time regions passed initial thresholding. Multiple values in a single cell correspond to multiple time regions that passed initial thresholding. Earlier time regions are listed first.

			***p*-value**		
	**Region**	***n***	**Day 1**	**Day 3**	**Day 4**
**GP1**	GP	238	**0.000100**	**0.000100**	**0.000100**
	Put	238	**0.002300**	**0.002500**	**0.000100**
	M1/S1	238	**0.015898**	**0.000100**	**0.000100**
	PM	238	**0.000500**	**0.000500**	**0.000900**
**GP2**	GP	202		0.155084	
	Put	202		**0.009099**	**0.000100**
	M1	202	**0.001400**	**0.000100**	**0.000300**
	PM	202	**0.003100**	**0.001700**	**0.004000**
**STN1**	vSTN	192		**0.000100**	**0.000100**
	dSTN	192		**0.000100**	**0.001200**
	M1	192	**0.045195**<br>0.130987	**0.042196**<br>**0.036096**	**0.008199**
	Par	192	0.120588<br>0.158384	**0.000100**	**0.000100**
**STN2**	vSTN	182	**0.034497**		0.098290
	dSTN	182			
	M1	182	**0.024198**	**0.021398**	**0.000100**
	Par	182	**0.000100**	**0.000100**	0.106189<br>0.078192

Supplementary Table 5. Across-day testing of local cue-related δ phase locking value: *p*-values. Blank cells indicate no time regions passed initial thresholding. Multiple values in a single cell correspond to multiple time regions that passed initial thresholding. Earlier time regions are listed first.

				***p*-value**	
	**Region**	***n***	**Day 1 vs. 3**	**Day 3 vs. 4**	**Day 1 vs. 4**
**GP1**	GP	238	**0.035096**		**0.002700**
	Put	238	0.195580<br>0.075092	0.082892	0.094291
	M1/S1	238	**0.0150985**<br>0.195780		**0.041896**
	PM	238			**0.002400**
**GP2**	GP	202			
	Put	202	**0.043596**		0.170983<br>0.170983
	M1	202	**0.020798**	0.074493	**0.044496**
	PM	202	**0.022298**	0.206479	
**STN1**	vSTN	192	**0.000100**		0.189781<br>**0.001500**
	dSTN	192	**0.000100**	**0.022098**	**0.001200**
	M1	192	0.108189	**0.032097**	
	Par	192	**0.000600**		**0.000100**
**STN2**	vSTN	182			
	dSTN	182			
	M1	182			
	Par	182			**0.017498**

Supplementary Table 6. Baseline testing of δ PPC: *p*-values.
Blank cells indicate no time regions passed initial thresholding. Multiple values in a single cell correspond to multiple time regions that passed initial thresholding. Earlier time regions are listed first.

				***p*-value**	
	**Region**	***n***	**Day 1**	**Day 3**	**Day 4**
**GP1**	GP-Put	238	**0.001400**	**0.001300**	**0.003200**
	GP-M1/S1	238	**0.016798**	**0.001800**	**0.007499**<br>0.124588
	GP-PM	238	**0.004500**	**0.000400**	**0.000400**
	Put-M1/S1	238	0.094291<br>**0.042696**	**0.005799**	**0.012799**
	Put-PM	238	**0.004100**	**0.004900**	**0.002000**
	M1/S1-PM	238	**0.002900**	**0.004100**	**0.000500**
**GP2**	GP-Put	202			
	GP-M1	202			
	GP-PM	202			
	Put-M1	202		**0.037796**	
	Put-PM	202	0.164084<br>0.106589		0.060794
	M1-PM	202		**0.004500**	**0.000600**
**STN1**	vSTN-dSTN	192		0.174083<br>**0.005999**	0.126987
	vSTN-M1	192		0.076692	0.054195
	vSTN-Par	192			**0.001900**
	dSTN-M1	192	0.180782	**0.010099**	**0.002100**
	dSTN-Par	192		**0.003400**	**0.000600**
	Par-M1	192		**0.000300**	**0.002400**
**STN2**	vSTN-dSTN	182	**0.000100**		**0.000100**
	vSTN-M1	182	**0.005699**		

vSTN-Par	182	0.130587		0.074693
dSTN-M1	182			
dSTN-Par	182	0.088791		0.111189<br>0.087591
Par-M1	182			

Supplementary Table 7. Across-day testing of δ PPC: *p*-values.

Blank cells indicate no time regions passed initial thresholding. Multiple values in a single cell correspond to multiple time regions that passed initial thresholding. Earlier time regions are listed first.

			p-value		
	**Region**	***n***	**Day 1 vs. 3**	**Day 3 vs. 4**	**Day 1 vs. 4**
**GP1**	GP-Put	476	0.121488	**0.022398**	0.089191
	GP-M1/S1	476			
	GP-PM	476	0.182882		0.073093
	Put-M1/S1	476	0.140886		0.075392
	Put-PM	476	0.105789	**0.028097**	0.092891
	M1/S1-PM	476			
**GP2**	GP-Put	404	**0.000100**	0.118988	**0.000100**
	GP-M1	404			
	GP-PM	404	**0.006199**		**0.028197**<br>**0.007699**
	Put-M1	404	**0.022098**	0.242376	0.208179
	Put-PM	404			0.067693
	M1-PM	404	**0.001700**		**0.000400**
**STN1**	vSTN-dSTN	384	**0.000100**	0.053395<br>0.095890	**0.000100**
	vSTN-M1	384		0.161384	
	vSTN-Par	384		**0.017998**	0.174183
	dSTN-M1	384	**0.020598**	0.130387	
	dSTN-Par	384	**0.005199**	0.197480<br>0.138986	**0.000600**
	Par-M1	384	0.185581		
**STN2**	vSTN-dSTN	364		0.086191	**0.000100**
	vSTN-M1	364	**0.006799**	0.115288	0.069793
	vSTN-Par	364			

dSTN-M1	364			
dSTN-Par	364			0.163184
Par-M1	364		0.131287	

Supplementary Table 8. Baseline testing of δ PSI: *p*-values.
Blank cells indicate no time regions passed initial thresholding. Multiple values in a single cell correspond to multiple time regions that passed initial thresholding. Earlier time regions are listed first. NA, not applicable.

			***p*-value**		
	**Region**	***n***	**Day 1**	**Day 3**	**Day 4**
**GP1**	GP-Put	238	0.081592<br>**0.000300**	**0.000100**	**0.000300**
	GP-M1/S1	238	**0.003400**	**0.001600**	**0.000800**
	GP-PM	238		**0.032897**	**0.005799**
	Put-M1/S1	238	**0.018798**	0.088191<br>**0.001300**	**0.001600**
	Put-PM	238	**0.001500**	**0.000800**	**0.000300**
	M1/S1-PM	238	**0.000400**	**0.000600**	**0.000600**
**GP2**	GP-Put	202	NA	NA	NA
	GP-M1	202	NA	NA	NA
	GP-PM	202	NA	NA	NA
	Put-M1	202	NA		NA
	Put-PM	202	NA	NA	NA
	M1-PM	202	NA	**0.000600**	0.089691
**STN1**	vSTN-dSTN	192	NA		NA
	vSTN-M1	192	NA	NA	NA
	vSTN-Par	192	NA	NA	
	dSTN-M1	192	NA	**0.004400**	0.059394
	dSTN-Par	192	NA		**0.012499**
	Par-M1	192	NA		**0.013399**
**STN2**	vSTN-dSTN	182		NA	
	vSTN-M1	182		NA	NA
	vSTN-Par	182	NA	NA	NA

dSTN-M1	182	NA	NA	NA
dSTN-Par	182	NA	NA	NA
Par-M1	182	NA	NA	NA

Supplementary Table 9. Across-day testing of session-wide null PPC: *p*-values.

	***p*-value**
**GP1**	**0.000100**
**GP2**	**0.000100**
**STN1**	**0.000100**
**STN2**	**0.000100**

Supplementary Table 10. Baseline testing of intraregional δ-γ_h^δ PPC: sequence learning-related *p*-values.

Blank cells indicate no time regions passed initial thresholding. Multiple values in a single cell correspond to multiple time regions that passed initial thresholding.

			p-value		
	**Region**	***n***	**Day 1**	**Day 3**	**Day 4**
**GP1**	GP	238			
	Put	238	0.284870		
	M1/S1	238		**0.000400**	**0.046695**
	PM	238		0.097890	0.203880
**GP2**	GP	202	0.19548<br>**0.034697**		
	Put	202			0.198180
	M1	202		0.297770<br>**0.001100**	0.058994
	PM	202		0.130990	
**STN1**	vSTN	192	0.184380		
	dSTN	192			0.304670
	M1	192		0.070593	**0.024998**
	Par	192	**0.041596**	0.166280	
**STN2**	vSTN	182			
	dSTN	182	**0.000100**	0.143390<br>**0.010499**	
	M1	182	**0.006999**	**0.000100**	**0.000100**
	Par	182		**0.040096**	**0.002900**

Supplementary Table 11. Across-day testing of intraregional δ-γ_h^{δ} PPC: sequence learning-related *p*-values.

Blank cells indicate no time regions passed initial thresholding. Multiple values in a single cell correspond to multiple time regions that passed initial thresholding.

			p-value		
	**Region**	***n***	**Day 1 vs. 3**	**Day 3 vs. 4**	**Day 1 vs. 4**
**GP1**	GP	476		0.228380	
	Put	476			
	M1/S1	476	0.050295	**0.015898**	
	PM	476			0.250570
**GP2**	GP	404			0.186080
	Put	404		0.102790	0.115090
	M1	404	0.186280<br>**0.000300**	0.277470	
	PM	404			
**STN1**	vSTN	384			0.110190
	dSTN	384			**0.008599**
	M1	384	0.081292	0.133390<br>**0.028397**	**0.034097**
	Par	384			
**STN2**	vSTN	364			
	dSTN	364			
	M1	364			
	Par	364			**0.006899**

Supplementary Table 12. Baseline testing of intraregional δ-γ_h^{δ} PSI: sequence learning-related *p*-values.

Blank cells indicate no time regions passed initial thresholding. Multiple values in a single cell correspond to multiple time regions that passed initial thresholding. Earlier time regions are listed first. NA, not applicable.

			p-value		
	**Region**	***n***	**Day 1**	**Day 3**	**Day 4**
**GP1**	GP	238	NA	NA	NA
	Put	238	NA	NA	NA
	M1/S1	238	NA	**0.001200**	**0.016998**
	PM	238	NA	NA	NA
**GP2**	GP	202	**0.021698**	NA	NA
	Put	202	NA	NA	NA
	M1	202	NA		NA
	PM	202	NA	NA	NA
**STN1**	vSTN	192	NA	NA	NA
	dSTN	192	NA	NA	NA
	M1	192	NA	NA	**0.031397**
	Par	192	**0.008699**<br>**0.026997**	NA	NA
**STN2**	vSTN	182	NA	NA	NA
	dSTN	182			NA
	M1	182		**0.000100**	
	Par	182	NA	**0.005599**	**0.017498**

Supplementary Table 13. Baseline testing of movement-related γ amplitude synchronization: *p*-values.

Blank cells indicate no time regions passed initial thresholding. Multiple values in a single cell correspond to multiple time regions that passed initial thresholding. For each statistical test, 10,000 bootstrap samples of gamma amplitude were generated. The p-value was computed as percent of bootstrap sample means < 0.

			***p*-value**		
	**Region**	***n***	**Day 1**	**Day 3**	**Day 4**
**GP1**	GP	238	0.884712	0.192081	0.956804
	Put	238	0.558444	0.238276	0.788321
	M1/S1	238	**0.000100**	**0.000100**	**0.000100**
	PM	238	0.120588	**0.009799**	**0.000100**
**GP2**	GP	206	0.308669	0.762524	0.695830
	Put	206	0.232177	0.936106	**0.047995**
	M1	206	**0.000100**	**0.000900**	**0.000100**
	PM	206	0.522548	0.770323	0.067493
**STN1**	vSTN	154	0.984202	0.202380	0.185681
	dSTN	154	0.996300	**0.021198**	0.627437
	M1	154	**0.006599**	**0.000100**	**0.001100**
	Par	154	0.853415	**0.000100**	**0.001100**
**STN2**	vSTN	184	0.317568	0.297170	0.198980
	dSTN	184	0.686131	0.794721	0.779622
	M1	184	**0.000100**	**0.000100**	**0.000100**
	Par	184	0.967303	0.647535	0.988501

Supplementary Table 14. Baseline testing of interregional δ-γ_h^δ PPC: sequence learning-related *p*-values.

Blank cells indicate no time regions passed initial thresholding. Multiple values in a single cell correspond to multiple time regions that passed initial thresholding.

			p-value		
	**Region**	***n***	**Day 1**	**Day 3**	**Day 4**
**GP1**	Put-M1/S1	238		0.194480<br>**0.002200**	0.178580
	Put-PM	238		**0.000500**	0.141290
	M1/S1-PM	238		**0.026197**	0.305570
	PM-M1/S1	238	0.051495<br>0.309370	**0.001500**	0.086391
**GP2**	Put-M1	202			
	Put-PM	202	0.137190		0.140690
	M1-PM	202	0.312570	0.072593	0.199380<br>0.299170
	PM-M1	202		**0.001500**	0.103290
**STN1**	vSTN-dSTN	192		**0.007399**	0.110590
	dSTN-M1	192	0.333870	**0.000100**	**0.002100**
	Par-M1	192	0.151680	**0.001700**	
**STN2**	vSTN-dSTN	182			
	dSTN-M1	182	**0.000100**		
	Par-M1	182	**0.000100**	**0.002700**	

Supplementary Table 15. Across-day testing of interregional δ-γ_h^δ PPC: sequence learning-related *p*-values.

Blank cells indicate no time regions passed initial thresholding. Multiple values in a single cell correspond to multiple time regions that passed initial thresholding.

			p-value		
	**Region**	***n***	**Day 1 vs. 3**	**Day 3 vs. 4**	**Day 1 vs. 4**
**GP1**	Put-M1/S1	238	**0.047695**<br>**0.002000**	0.223680<br>**0.005400**	0.192780
	Put-PM	238	0.060894<br>0.051795	0.051095<br>0.152480	
	M1/S1-PM	238	0.050395		
	PM-M1/S1	238	**0.008599**	**0.007899**	
**GP2**	Put-M1	202			
	Put-PM	202	0.267070		0.257170
	M1-PM	202			
	PM-M1	202	0.069393		
**STN1**	vSTN-dSTN	192	**0.003200**		
	dSTN-M1	192	**0.001100**	**0.000800**	0.076792<br>0.136990
	Par-M1	192	**0.004600**	**0.011999**	
**STN2**	vSTN-dSTN	182			
	dSTN-M1	182			
	Par-M1	182			**0.000100**

Supplementary Table 16. Baseline testing of interregional δ-γ_h^{δ} PSI: sequence learning-related *p*-values.

Blank cells indicate no time regions passed initial thresholding. Multiple values in a single cell correspond to multiple time regions that passed initial thresholding. Earlier time regions are listed first. NA, not applicable.

				***p*-value**	
	**Region**	***n***	**Day 1**	**Day 3**	**Day 4**
**GP1**	Put-M1/S1	238	NA	**0.005399**	NA
	Put-PM	238	NA	**0.025097**	NA
	M1/S1-PM	238	NA	**0.031497**	NA
	PM-M1/S1	238	NA	**0.006699**	NA
**GP2**	Put-M1	202	NA	NA	NA
	Put-PM	202	NA	NA	NA
	M1-PM	202	NA	NA	NA
	PM-M1	202	NA		NA
**STN1**	vSTN-dSTN	192	NA	0.105989<br>**0.043797**	NA
	dSTN-M1	192	NA	**0.006399**	**0.047595**
	Par-M1	192	NA	**0.022698**	NA
**STN2**	vSTN-dSTN	182	NA	NA	NA
	dSTN-M1	182		NA	NA
	Par-M1	182	**0.048195**	**0.021898**	NA

Supplementary Table 17. Baseline testing of movement-related β amplitude desynchronization: *p*-values.

Blank cells indicate no time regions passed initial thresholding. Multiple values in a single cell correspond to multiple time regions that passed initial thresholding.

			***p*-value**		
	**Region**	***n***	**Day 1**	**Day 3**	**Day 4**
**GP1**	GP	238	**0.000100**	**0.000100**	**0.000100**
	Put	238	**0.000900**	**0.000100**	**0.000300**
	M1/S1	238	**0.000100**	**0.000100**	**0.000100**
	PM	238	**0.000100**	**0.000100**	**0.000100**
**GP2**	GP	206	0.518448	**0.002800**	0.839816
	Put	206	**0.000100**	**0.000300**	**0.001000**
	M1	206	**0.000100**	**0.000100**	**0.000100**
	PM	206	**0.000100**	**0.000500**	**0.000100**
**STN1**	vSTN	154	0.072893	0.128887	0.230977
	dSTN	154	**0.001900**	**0.017898**	**0.009299**
	M1	154	**0.000100**	**0.000100**	**0.000100**
	Par	154	**0.004400**	**0.000100**	**0.000100**
**STN2**	vSTN	184	0.878312	0.796820	0.958004
	dSTN	184	0.200280	0.142286	0.298270
	M1	184	**0.000600**	**0.003700**	**0.000100**
	Par	184	0.931507	0.510249	0.300370

Supplementary Table 18. Baseline testing of intraregional δ-β^{δ} PPC: sequence learning-related *p*-values.

Blank cells indicate no time regions passed initial thresholding. Multiple values in a single cell correspond to multiple time regions that passed initial thresholding

			p-value		
	**Region**	***n***	**Day 1**	**Day 3**	**Day 4**
**GP1**	GP	238	0.078792<br>**0.007699**	**0.002000**	**0.000100**
	Put	238	0.101590	0.137790	
	M1/S1	238	**0.008199**<br>0.122790	**0.000100**	0.082492
	PM	238	**0.011399**	**0.002400**	**0.006799**
**GP2**	GP	202	**0.013999**	0.227380<br>0.175280	
	Put	202	0.066593		**0.048995**
	M1	202		**0.000200**	**0.017298**
	PM	202	**0.019398**	0.060294	0.154080
**STN1**	vSTN	192			
	dSTN	192			**0.015498**
	M1	192		**0.000500**	**0.001600**
	Par	192			**0.002300**
**STN2**	vSTN	182			
	dSTN	182	**0.000100**	**0.027397**	**0.000100**
	M1	182	0.134290<br>**0.015798**	0.085591	**0.004100**
	Par	182			

Supplementary Table 19. Across-day testing of intraregional δ-β$^{\delta}$ PPC: sequence learning-related *p*-values.

Blank cells indicate no time regions passed initial thresholding. Multiple values in a single cell correspond to multiple time regions that passed initial thresholding.

			p-value		
	**Region**	***n***	**Day 1 vs. 3**	**Day 3 vs. 4**	**Day 1 vs. 4**
**GP1**	GP	476		**0.045295**	**0.025497**
	Put	476			
	M1/S1	476	0.153880	**0.033597**	**0.009299**
	PM	476	0.212180		
**GP2**	GP	404			
	Put	404			0.244980<br>**0.006199**
	M1	404	**0.003300**	**0.000400**	0.239280<br>0.158480
	PM	404		0.220780	
**STN1**	vSTN	384			
	dSTN	384		0.193880	
	M1	384			
	Par	384			**0.005699**
**STN2**	vSTN	364			
	dSTN	364			
	M1	364			
	Par	364			

Supplementary Table 20. Baseline testing of intraregional δ-β$^{\delta}$ PSI: sequence learning-related *p*-values.

Blank cells indicate no time regions passed initial thresholding. Multiple values in a single cell correspond to multiple time regions that passed initial thresholding. Earlier time regions are listed first. NA, not applicable.

			p-value		
	**Region**	***n***	**Day 1**	**Day 3**	**Day 4**
**GP1**	GP	238	**0.005799**		**0.008199**
	Put	238	NA	NA	NA
	M1/S1	238		0.059694<br>**0.014699**	NA
	PM	238	0.080892		0.096390
**GP2**	GP	202		NA	NA
	Put	202	NA	NA	
	M1	202	NA		**0.001200**
	PM	202	**0.028397**<br>**0.015998**	NA	NA
**STN1**	vSTN	192	NA	NA	NA
	dSTN	192	NA	NA	
	M1	192	NA	**0.000100**	**0.000300**
	Par	192	NA	NA	0.067693
**STN2**	vSTN	182	NA	NA	NA
	dSTN	182			
	M1	182		NA	0.060394
	Par	182	NA	NA	NA

Supplementary Table 21. Baseline testing of interregional δ-β^{δ} PPC: sequence learning-related *p*-values.

Blank cells indicate no time regions passed initial thresholding. Multiple values in a single cell correspond to multiple time regions that passed initial thresholding.

			***p*-value**		
	**Region**	***n***	**Day 1**	**Day 3**	**Day 4**
**GP1**	Put-M1/S1	238	**0.002300**	**0.001100**	**0.005799**
	Put-PM	238	**0.025697**	**0.003700**	**0.027197**
	M1/S1-GP	238	0.108990		**0.013499**
**GP2**	Put-M1	202		**0.000500**	0.137590<br>0.066093
	Put-PM	202		0.075092	0.148190
	M1-GP	202	0.238080	**0.000500**	**0.042296**

Supplementary Table 22. Across-day testing of interregional δ-β[δ] PPC: sequence learning-related *p*-values.

Blank cells indicate no time regions passed initial thresholding. Multiple values in a single cell correspond to multiple time regions that passed initial thresholding.

	Region	*n*	*p*-value<br>Day 1 vs. 3	Day 3 vs. 4	Day 1 vs. 4
**GP1**	Put-M1/S1	238	**0.016398**	**0.005500**	
	Put-PM	238	**0.028997**	**0.006799**	
	M1/S1-GP	238			
**GP2**	Put-M1	202	0.175780		0.129790
	Put-PM	202		**0.033297**	
	M1-GP	202	0.118190		

Supplementary Table 23. Baseline testing of interregional δ-β^{δ} PSI: sequence learning-related *p*-values.

Blank cells indicate no time regions passed initial thresholding. Multiple values in a single cell correspond to multiple time regions that passed initial thresholding. Earlier time regions are listed first. NA, not applicable.

			p-value		
	**Region**	***n***	**Day 1**	**Day 3**	**Day 4**
**GP1**	Put-M1/S1	238	**0.046895**	**0.002900**	
	Put-PM	238	**0.012599**	**0.006699**	
	M1/S1-GP	238	NA	NA	
**GP2**	Put-M1	202	NA		NA
	Put-PM	202	NA	NA	NA
	M1-GP	202	NA	**0.006599**	0.057394

Supplementary Table 24. Baseline testing of interregional δ-β$^{\delta}$ PPC: task exposure-related *p*-values.
Blank cells indicate no time regions passed initial thresholding. Multiple values in a single cell correspond to multiple time regions that passed initial thresholding.

			p-value		
	**Region**	***n***	**Day 1**	**Day 3**	**Day 4**
**GP1**	GP-M1/S1	238	**0.002600**	**0.000100**	**0.000400**
	GP-PM	238	**0.001800**	**0.000100**	**0.000300**
	Put-GP	238	**0.001400**	**0.004600**	**0.024698**
	PM-M1/S1	238	**0.020798**	**0.000300**	**0.000200**
**GP2**	GP-M1	202			**0.043396**
	GP-PM	202			
	Put-GP	202	**0.031797**<br>0.236980<br>0.231280		
	PM-M1	202	**0.018498**	**0.001000**	**0.020098**
**STN1**	M1-Par	192			**0.002600**
	Par-dSTN	192			**0.006799**<br>0.100790
**STN2**	M1-Par	182	0.110690		
	Par-dSTN	182			

Supplementary Table 25. Across-day testing of interregional δ-β^δ PPC: task exposure-related *p*-values.

Blank cells indicate no time regions passed initial thresholding. Multiple values in a single cell correspond to multiple time regions that passed initial thresholding.

			p-value		
	**Region**	***n***	**Day 1 vs. 3**	**Day 3 vs. 4**	**Day 1 vs. 4**
**GP1**	GP-M1/S1	238	**0.036996**<br>**0.004900**	0.137690	0.110190
	GP-PM	238			
	Put-GP	238		0.131690	
	PM-M1/S1	238	**0.000200**		**0.000400**
**GP2**	GP-M1	202			**0.010399**
	GP-PM	202	0.240780		0.215780
	Put-GP	202			
	PM-M1	202	0.192780		
**STN1**	M1-Par	192		0.058994	**0.004100**
	Par-dSTN	192		0.130390<br>0.156380	**0.004600**<br>0.140990
**STN2**	M1-Par	182	0.157680		0.135390
	Par-dSTN	182			

Supplementary Table 26. Baseline testing of interregional δ-β^{δ} PSI: task exposure-related *p*-values.

Blank cells indicate no time regions passed initial thresholding. Multiple values in a single cell correspond to multiple time regions that passed initial thresholding. Earlier time regions are listed first. NA, not applicable.

			***p*-value**		
	**Region**	***n***	**Day 1**	**Day 3**	**Day 4**
**GP1**	GP-M1/S1	238	0.079892		**0.002200**
	GP-PM	238			**0.000400**
	Put-GP	238		**0.005699**	**0.018698**
	PM-M1/S1	238		0.057894	**0.028197**
**GP2**	GP-M1	202	NA	NA	
	GP-PM	202	NA	NA	NA
	Put-GP	202		NA	NA
	PM-M1	202		**0.028497**	**0.000300**
**STN1**	M1-Par	192	NA	NA	**0.006099**
	Par-dSTN	192	NA	NA	**0.001500**
**STN2**	M1-Par	182	NA	NA	NA
	Par-dSTN	182	NA	NA	NA
